# A Functional Contextual Account of Background Knowledge in Categorization: Implications for Artificial General Intelligence and Cognitive Accounts of General Knowledge

**DOI:** 10.3389/fpsyg.2022.745306

**Published:** 2022-03-02

**Authors:** Darren J. Edwards, Ciara McEnteggart, Yvonne Barnes-Holmes

**Affiliations:** ^1^Department of Public Health, Policy, and Social Sciences, Swansea University, Swansea, United Kingdom; ^2^Department of Experimental Clinical and Health Psychology, Ghent University, Ghent, Belgium

**Keywords:** functional contextualism, machine learning, Relational Frame Theory (RFT), categorization, background knowledge

## Abstract

Psychology has benefited from an enormous wealth of knowledge about processes of cognition in relation to how the brain organizes information. Within the categorization literature, this behavior is often explained through theories of memory construction called exemplar theory and prototype theory which are typically based on similarity or rule functions as explanations of how categories emerge. Although these theories work well at modeling highly controlled stimuli in laboratory settings, they often perform less well outside of these settings, such as explaining the emergence of background knowledge processes. In order to explain background knowledge, we present a non-similarity-based post-Skinnerian theory of human language called Relational Frame Theory (RFT) which is rooted in a philosophical world view called functional contextualism (FC). This theory offers a very different interpretation of how categories emerge through the functions of behavior and through contextual cues, which may be of some benefit to existing categorization theories. Specifically, RFT may be able to offer a novel explanation of how background knowledge arises, and we provide some mathematical considerations in order to identify a formal model. Finally, we discuss much of this work within the broader context of general semantic knowledge and artificial intelligence research.

## Introduction

Category learning has been described as fundamental to all aspects of decision-making, and refers to the process of organizing sensory experience into groups and appears to be key to understanding the world (Margolis and Laurence, 1999; Lakoff, 2008). The main purpose of the human cognitive system for developing concepts and categories is cognitive economy, which allows individuals to process complex information in a manageable way in spite of finite memory storage (Goldstone et al., 2018). Categorization researchers use models to describe the process of categorization in a formal (and sometimes mathematical), principled and lawful way, in an attempt to achieve prediction over behavior in particular categorization tasks (Pothos and Wills, 2011).

Categorization processes can be separated into four distinct areas of research, supervised categorization (Nosofsky, 1988; Minda and Smith, 2001; Hampton, 2007; Kurtz, 2007; Vanpaemel and Storms, 2008) and unsupervised categorization (Fleming and Cottrell, 1990; Ashby et al., 1999; Pothos and Chater, 2002; Pothos and Close, 2008; Pothos et al., 2011) which can utilize a similarity or rule based function. There is also the emerging area of categorization called relational representation (Stewart et al., 2005; Edwards et al., 2012) which explores inference learning in categorization, and also an area known as background knowledge (Heit, 2001) which attempts to incorporate many of the above approaches to understand how background knowledge emerges and affects category decision making.

Background knowledge in categorization refers to the beliefs that individuals may have about the interrelations and causal connections among features and concepts which emerge through prior learning, and how this affects category decision making (Keil, 1989; Murphy, 1993). This differs in some ways from other approaches which attempt to model knowledge such as theories in semantics, for example semantic memory. Semantic memory can be regarded as a category of long-term memory which involves the recollection of ideas, concepts, and facts commonly regarded as general knowledge (Zesch et al., 2008; Paradis, 2012; Russo et al., 2021). However, the focus within semantics has primarily been on natural language, such as the logical relations within sentence structures which give meaning to the language being expressed (Löbner et al., 2021) (however there is modest overlap – see the section on “The limited success of semantics” for a more in depth overview). Crucially, background knowledge in categorization, does not specifically and primarily explore logical relations within linguistics (as semantics does), but instead, focuses primarily on the category structure developed because of prior learning which helps form some general knowledge about some concept, and facilitates some category decision in the present moment.

In an example of background knowledge, if you were to ask a lay person (non-animal specialist) to describe the categorical features of a bird and a bat, they may respond by saying; “Birds and bats fly by using their wings to do so. A bat prefers to fly at night, whilst a bird prefers to fly in the day. Birds has feathers whilst bats do not.” This relies on prior knowledge learned perhaps in early school, through parents and general books the person may have read out of interest. Causal connections and interrelations then are drawn by the participant from this knowledge in order for them to describe how wings are used to allow the bird and bat to fly. Specific differences are also identified by the participant such as when birds and bats prefer to fly, but little causal connections are drawn by the lay person as they lack the essential background knowledge of why this may be the case. However, if you were to ask an animal specialist, they may add to this by saying; “A bat uses echolocation to fly at night, to identify food, and to navigate, whilst a bird requires light to fly and does not use echolocation. A bat is a mammal, and feeds on milk from its mother while growing, whilst a bird is a member of the Aves, and is not a mammal.” This specialist background knowledge may have been learned during higher education and allows the specialist to draw stronger and more accurate causal interrelations and connections amongst the feature of birds and bats. The study of background knowledge, is thus to identify how these causal connections and interrelations between concepts develop, in what context they emerge, and to identify the different ways in which this knowledge can influence categorization behavioral decisions in a given categorization task (Heit, 2001).

In this current review and conceptual development paper, we (1) firstly broadly explain why existing categorization models fail to account for background knowledge. We also briefly highlight why the problem of background knowledge is also a problem in artificial intelligence (AI) research, for those who seek to develop artificial general intelligence (AGI), and for which development in that area may be dependent on a model for background knowledge. We, therefore, seek this literature for clues of how we may develop mathematical models to solve the problem of background knowledge which may not have been considered previously; (2) We offer a functional contextual approach to understanding background knowledge which has not yet been considered in background knowledge research; (3) Finally, as part of this exploration, we offer a formal mathematical model of this functional contextual approach for use with Background knowledge experiments, which is consistent with the approach made by many other researchers who offer mathematical accounts of their categorization models, and for which may be of interest to categorization as well as AI mathematical modelers. This takes into consideration the modeling of both similarity as well as functional contextual properties within a RFT framework. We then make suggestions for future work, which specifically test the descriptive and predictive power of this RFT approach for background knowledge in categorization.

## The Problem of Background Knowledge and Why Existing Modeling Efforts Are So Far Incomplete

The area of background knowledge has been the most difficult for categorization researchers to formalize a specific model, and most attempts have only provided an intuitive account of this thus far (Heit, 1997; Dreyfus et al., 2000; Pothos and Wills, 2011) with most efforts in this area having now been redirected to less difficult problems such as simple induction (inference) modeling (B. K. Hayes and Heit, 2013; Hawkins et al., 2016). Indeed, approaches based on similarity (i.e., identifying similar features when categorizing) which have been used to explain behavior in unsupervised and supervised tasks have not provided an effective means of explaining the emergence of background knowledge though some promising progress has been made in relation to understanding background knowledge effects on categorization decisions.

Supervised learning models are often explained by exemplar and prototype theories which involve matching through similarity (magnitudes of length, height, color, etc.), either the individual memory trace of exemplars for the to-be categorized novel stimuli, or matching a prototype (e.g., a prototypical average representation of all chairs represented in memory) to the to-be categorized novel stimuli. This typically involves modeling how stimuli correspond to points in multidimensional space (usually Euclidean), and some similarity function is defined based on some mathematical axioms about how the stimuli should be categorized on this similarity basis (Lamberts and Shapiro, 2002; Pothos and Wills, 2011).

Similarity approaches are one of the earliest and most successful explanations of how people categorize (Posner and Keele, 1968; Reed, 1972; Rosch and Mervis, 1975; Pothos and Wills, 2011). In this account, the more similar item X is to what is known about category A (e.g., the magnitude of size), the more likely X will be categorized as belonging to A. Consider, for example, the classification of a novel bird. According to one similarity model called the prototype account (Rosch and Mervis, 1975), the reasoner would take notice of typical features such as color, wing-span, type of beak, and where the bird lives. They would then categorize the bird on these bases as belonging to a particular bird category if this bird had similar features to the prototypical list of features identified in that category (e.g., similar shaped beak, sized wing, etc.).

In typical similarity tasks, such as unsupervised categorization, participants are asked to put several items into sets of categories that they feel to be most intuitive. Crucially, the participant is not informed of any category structure, thus cannot infer from pre-existing knowledge about category structure. These unsupervised tasks are thought to involve category coherence (Rosch and Mervis, 1975; Murphy and Medin, 1985), which allow researchers to hypothesize about how intuitive the structures being categorized are, and how participants process this information without any background knowledge of what the category structure should look like, in order to form distinct categories. So, in this case, unsupervised categorization may be less helpful in facilitating researchers to identify a model of background knowledge, as these tasks typically do not require the use of it.

In supervised (also a similarity based account) categorization tasks (Nosofsky, 1988; Hampton, 2007), participants are shown the exemplars of each category (such as 20 pictures of two novel creatures) in addition to a category label (such as Blib or Chomp), and thus learn the category structure. They are then asked to decide which category a set of new items (pictures of new creatures) belong to (either the Blib or Chomp creature category). Hence, unlike unsupervised tasks, supervised tasks involve some aspect of learning about the category structure before the categorization decisions are made, and the categorization process relies of these previous memory traces of the category structure when making category decisions. This, therefore, at a conceptual level may be more helpful in facilitating researchers to identify a model of background knowledge, as these tasks require the use of memory trace, and background knowledge presumably would need to be remembered via such a memory trace. This is because, if a categorization task asks the participants, ‘is a bat and a bird in the same category?’, their answer should be dependent on some memory trace of background knowledge that a “bat is a mammal, whilst a bird is not,” therefore the participants come to the conclusion that they are not in the same category. Without such a memory trace, the participant may determine that a bat and bird are in the same category on the basis of some more general similarity function based on the shape and size similarity of the creatures (i.e., similar to an unsupervised approach which does not require a strong memory trace).

In both supervised and unsupervised categorization tasks, many models have been formalized mathematically, and are typically based on a similarity axiom. For example, the Generalized Context Model (GCM; Nosofsky, 1986) is a generalization of the context model by Medin and Schaffer (1978), integrated with similarity and choice of classical theories by Garner (1974). It is also one of the most heavily cited and influential models in categorization research (over 3200 citations). The GCM incorporates multidimensional scaling (MDS) to model similarity, whereby multidimensional space is used to represent the exemplars and the similarity is a decreasing function of their distance in this space. There are, of course, many other similarity-based models, and a detailed examination of these is beyond of the scope of this current article. One example of an unsupervised categorization model is the simplicity model which predicts the “optimal” categories through an information reductionism perspective (Pothos and Chater, 2002; Nikolopoulos and Pothos, 2009). This model assumes that information theory applies to cognition through a simplicity principle, which states that we tend to prefer simpler and not more complex perceptual organizations. There are also models, such as the rational model (Anderson, 1990) which use features in their description of a categories should emerge. This takes the dimensional features (e.g., has four legs, barks, and has tail) and identifies the probability of which category the item belongs to, based on how similar its features are to the features within each of the categories (where assignment is made with the greatest similarity).

However, criticisms of these similarity models as a basis for natural concepts came as far back as from Murphy and Medin (1985) in a seminal paper concerning knowledge effects on concept learning. Rips (1989) then extended this criticism by reporting a number of cases whereby categorization behavior was better explained by a rule based account as opposed to a similarity-based account. For example, he highlighted the pizza coin experiment, where participants were asked to imagine an item that was halfway between two categories (a pizza and a coin), where one of the categories had fixed magnitudes of properties (such as fixed size – coin) and the other had variable magnitudes of properties (such as variable size – pizza). Participants were then asked: (1) whether an item was more similar to one of the categories than the other, and (2) whether an item was more likely to be a member of one category rather than the other. Rips found that participants were more likely to categorize the imagined item as belonging to the variable category (pizza) but more similar to the fixed category (coin). Rips concluded that there was sometimes a disassociation between similarity and categorization, i.e., individuals categorization behavior were not always consistent with how similar they felt items were with category members.

Perhaps in an even more convincing example, Rips (1989) asked participants to imagine a creature which accidentally metamorphosized from one category (bird) to another (insect). When asked whether the creature was more similar to a bird or an insect, most participants selected insect, and yet they more readily categorized the creature as a bird. In a separate condition, some participants were asked to judge the similarity of the creature before it transformed and to assume that metamorphosis happened naturally. In this condition, the participants deemed the creature more similar to a bird, and this finding suggested that some background knowledge about essential qualities (e.g., DNA) were being inferred from the detail of metamorphosis and the extent to which this was a natural process. Again, Rips proposed that this was evidence that participants were using formal rules of inference from background knowledge to inform their categorization decisions, and not merely similarity.

So, in spite of the success of similarity models in predicting categorization behavior based on essential category structure in many supervised and unsupervised tasks, they appear less effective in predicting behavior in tasks which involve a rules option and involve inferences made through background knowledge. The view that in addition to perceptual similarity, rule, and background knowledge information pertaining to the stimulus context may also be relevant, represented a shift away from similarity-based explanations, and toward the inclusion of critical features (Katz, 1972; Komatsu, 1992). In this classic view of critical features, necessary or sufficient features were deemed important in categorization that involved generalizable background knowledge.

In an example of critical features, consider the statement “not coming from Mars” as a necessary feature of the concept “human,” which can only be derived through background knowledge context (i.e., humans do not come from Mars). However, this classical theory may also be limited as many non-humans also do not come from mars, so the classification of “necessary” may be overly simplistic. In another example, the concept “man” is a necessary feature of “bachelor,” however, some men are not bachelors. So, again, utilizing the simple idea of a “necessary” feature is clearly limited. Indeed, one of the main problems with the naïve theory approach, is that the formalization of background knowledge into a specific model has been shown to be very difficult. As a result of this, only an intuitive account of background knowledge has been achieved through the cognitive categorization approach (Heit, 1997; Dreyfus et al., 2000; Pothos and Wills, 2011). The difficulties of tackling the very complex problem of background knowledge as a formal model, had led categorization researchers to return to similarity models and rule-based explanations to explain some of the simpler elements of knowledge effects on the categorization process.

Neural (connectionist) networks have also been used to model how people categorize stimuli, which utilize learned weighted associations between features in order to make classifications. One example of this is the competitive learning feature detector neural network (Rumelhart and Zipser, 1985) for unsupervised categorization. Another example is the adaptive resonance theory (ART) model, which is based on the stability-plasticity dilemma (Carpenter and Grossberg, 1988). The dilemma refers to the problem of how a learning system can remain adaptive or plastic in response to significant events and can remain stable against irrelevant events. The mathematical model uses a self-organizing neural network which organizes arbitrary input patterns in real time.

Though all of these models provide some basis for exploring how background knowledge can affect a categorization task, it is acknowledged these models are incomplete, as the models do not specify a framework which can be used in order to specify the relevant information (B. K. Heit, 1997; Hayes and Heit, 2013; Hawkins et al., 2016). This incompleteness stems in what is generally referred to by Heit as the knowledge selection problem, and it specifically relates to the problem, whereby, though the models discussed can address the processes in which background knowledge and new information are combined, they cannot address the processes for how a learner dynamically determines which background knowledge is most relevant, how this knowledge is generated, which context are important, and how causal connections and interrelations amongst concepts emerge within learning. As an example of this, consider Heit (1994) example of learning about joggers. In this study, and a follow up study (Heit, 1995), Heit demonstrated that information at various times points were being inferred and integrated, whereby new knowledge was derived from simple combinations of background knowledge and observations. However, this was a simple observation about the integration of knowledge, and was without a precise formal model to specify how exactly such information was being integrated and within what specific context.

Heit (1994), had made specific observations that when participants were asked to categorize whether people in a new city were joggers, background knowledge was assumed to be used about joggers in a previous city. While this assumption may be straightforward in a laboratory context, real life contains an almost infinite number of possibilities for knowledge selection, such as cultural background, occupation, hobbies, prior city experience, etc. This makes the knowledge selection problem very difficult to circumvent.

One possible way forward which has been proposed is to explore knowledge inference and induction more formally, as Heit (1997) suggested, which comes from the reasoning and memory literature (Anderson, 1991; Ross and Murphy, 1996), and may give us some clues of how to progress theories in background knowledge for categorization, albeit still not a complete model. When utilizing reasoning in the form of induction, for example, you may expect that if you learn that a person belongs to a category of “salespersons” you may then infer that this person will try to sell you something. This of course is missing the importance of context, as a “salesperson” is only likely to sell you something in the context of the place where they work. So, any complete model would need both induction and sensitivity to context.

Induction tasks are designed to answer questions about how participants can draw inferences for the information provided and when using background knowledge. A simple example of this would be asking a participant; ‘Crows are likely to contract a disease, how likely is it that robins will contract a disease?’ If the participant answers that it is likely that the robin will also contract the disease, this indicates that knowledge about the similarity between robins and crows may have been inferred.

Variations of this type of inductive reasoning task have shown that there are two kinds of inductive information which are important for understanding how background knowledge is generated and utilized within categorization tasks (Rips, 1975; Osherson et al., 1990). The first relates to the suggestion that when the premise category (e.g., crow) and the conclusion category (e.g., robin) are more similar, the induction inferences will have a stronger effect. The second is that lower rather than higher variability in the category leads to stronger inferences (Nisbett et al., 1983). In order to model these influences of inferences in category knowledge a category-based induction (CBI) model was developed (Osherson et al., 1990) which addresses some of these effects.

Another effect, called the selective weighting effects, has also been suggested as evidence of inductive reasoning. Here, focusing on certain features of the categories based on background knowledge has been suggested to be important (Heit and Rubinstein, 1994). For instance, consider this argument being presented to a participant: ‘Robins travel shorter distances in the extreme heat. How likely is it that bats travel shorter distances in extreme heat?’ In this case participants focused on the behavioral property “to travel” when comparing categories of robins and bats. As bats and robins are similar in that they both fly, then most participants inferred that bats and robins would be similar in the distance of travel when given extreme heat conditions. However, when asked a different way: ‘Robins have livers with two chambers. How likely is it that bats have livers with two chambers?’ Now the feature in comparing categories of bats with robins was focused on the “anatomy.” As bats are mammals and robins are birds, participants inferred that it was less likely that bats and robins would be similar in terms of anatomy (two chambers in their liver). This selective weighting implies that knowledge selection is based on context, in this case functional properties of flying and anatomy.

Other more applied areas to consider, in for example the social domain (social categories), relate induction within the area of social psychology and relational memory, such as the influence of social stereotypes and schema which are social categories. Stangor and Ruble (1989) demonstrated that congruent commercials (girls playing with doll) were recalled better than incongruent commercials (girls playing with trucks). This may represent some important areas for applied work in the future, where a background knowledge model could help to identify relevant functional processes within the background knowledge which need to be targeted by some social intervention in order to remediate these types of stereotypes.

However, although these models are very encouraging, and have demonstrated that background knowledge affects category learning, where inductive learning and memory have been identified as important in this process, these models have still been suggested to be incomplete. Heit (1997) suggested that their incompleteness relates to the need for more complex conceptions of representation outside of just exemplars, prototype, and rule based models. From this perspective, he suggests, a multi-modal representational scheme which accounts for both the relations among categories and the knowledge at multiple levels of abstraction, is needed, rather than overly focusing on whether one model (e.g., exemplar) provides a slightly better fit than another (e.g., prototype).

More recently in the last twenty years, specific modeling efforts for background knowledge categorization tasks have progressed very little. The more recent efforts of Heit and others have been to largely focus on similar problems of induction in categorization but more generally, and tweaking these types of models under different situations. For example, in one relatively recent study (B. K. Hayes and Heit, 2013), it was demonstrated that memory recognition shared some properties of induction. These category-based inductive inference models involve using the relations between categories to predict how individuals generalize novel properties of category exemplars, and reach conclusions that are likely but not certain given the available information. Jern and Kemp (2009) also showed that induction was involved in semantic repository, generalization, discovery, and identification, in relation to specific categorization tasks. The study identified, for example, that generalization was a problem for both supervised and unsupervised tasks, but unsupervised tasks also included the problem of relation discovery. Heit and colleagues have also explored how inferences are made over time, through the development of the Dynamic model for reasoning and memory (Hawkins et al., 2016). Through this model and corresponding data, they demonstrated that sequential sampling based on exemplar similarity and combined with hierarchical Bayesian analysis provided a more informative analysis in terms of the processes involved in inductive reasoning than the examination of choice data alone could provide.

So, induction and context seem to be an important avenue to further develop models for background knowledge. Unfortunately, however, many of the studies have focused on easier and more solvable problems such as congruent vs. incongruent tasks, or simple inference tasks. This perhaps falls short of what Heit had previously suggested, about the need for a multi-modal representational scheme which accounts for both the relations among categories and the knowledge at multiple levels of abstraction (Heit, 1997). Although these offer a useful starting point, they appear to tell us little about how or why knowledge is selected, i.e., they fail to solve knowledge selection problem. Developing a model which helps to understand what context knowledge is selected, or how the knowledge dynamically develops at a multi-modal representation level, and connects within a network of inference to allow for multiple levels of abstraction, are all important details in answering this problem.

In order to achieve this goal, a more general and holistic approach may be preferred, perhaps with new and fresh perspectives outside of traditional cognitive science approaches, and with a different ontological approach altogether. This approach should also capture a multi-modal representational scheme which will allow it to model multiple levels of abstraction and context. This should allow for greater ability to model more complex scenarios outside of simple congruence and inference tasks, such as how we make complex category decisions in the real world. For this, we propose considering other perspectives (in line with Heit’s suggestion) outside of the usual memory trace based exemplar, and prototype domain, and more in line with his recent work of category induction. In order to do this, we propose exploring a comprehensive functional contextual account which considers inference in the form of derived relations. In addition to this, this will include multiple modes of non-arbitrary similarity functions and arbitrary non-similarity learned contextual functions in order to account for categorization learning which uses background knowledge processes, and which may be able to offer some greater insights into solving the knowledge selection problem through modeling contextual learning more comprehensively.

As such, one approach may be to focus on functions and equivalent classes more concretely, and in a more formalized way in the form of *functional contextualism*. Cognitivism is based on the theorizing about mental representation, where memory trace, attention, inference between exemplar representations, etc., are specified and highlighted. In this way cognitivism in categorization can be thought of as a philosophy of science consistent with the ontology of realism which is a phenomenological paradigm, and which assumes that much of our perceptual reality exists based on the language and concepts which our cognitive system produces (Zahidi, 2014). Functional contextualism, on the other hand, is based on the ontology of pragmatism, and contextualism (Pepper, 1942; David and Mogoase, 2015) which instead of mental representation, it emphasizes the importance of what specific factors predicts and influences emotion, thoughts and behavior (decision making) which include categorization tasks. Crucially, it identifies the context in which the function of concepts, stimuli, thoughts, etc., occur, and how these exert different control on behavior and decision-making, with an emphasis on how an organism interacts with historically and situationally defined contexts in order to explain how background knowledge emerges and influences category decisions.

Through this functional-analytic approach some of the very specific problems with the knowledge selection problem may be overcome. This is because ontologically the cognitive mechanism approaches largely try to define the form of the environment through similarity and rule based approaches. This contrasts with a functional contextual approach which tries to define the context in which stimuli exert some functional control over the behavior and decision processes of the individual – hence enabling a more structured way to model learning in context and therefore enabling greater predictive and descriptive power over subtle contextual dependencies in which inference learning arises. These specific conceptual and ontological differences (form vs. function) may be key to resolving the knowledge selection problem, as categorization decision making which is dependent on background knowledge may be largely defined by our contextualized learning histories which are specific to each individual given their experiences.

Functional contextualism, therefore, may be helpful in providing such a philosophical foundation for modeling and formalizing an account of background knowledge by incorporating functions and the context of environmental stimuli at a holistic level, and more concretely than previous modeling attempts through inference (derived relation) type processes. This approach focuses on functional properties (such as functional equivalence) and contextual cues. For example, in an example of functional equivalence by Sidman (1994), given the category of cutlery, forks, and knives may be grouped together because of the background knowledge that these items share the functional property “to eat with.” However, in a different context, a knife may be used to peel paint off a wall (if you did not have a proper paint stripper tool) or defend yourself if you were attacked in the most extreme setting. Thus, the function is the purpose or use of that concept within a particular contextual setting. However, this early functional equivalence approach developed from Sidman (1994) has matured into a more formal model today. This approach is now embedded within the philosophical world view of functional contextualism, which defines that functions are dependent on context and specifically constitutes a post-Skinnerian behavior-analytic account of language known as Relational Frame Theory (RFT) (Barnes-Holmes et al., 2001; Blackledge, 2003; Torneke, 2010; De Houwer, 2013).

This fits well within the cognitive literature, as researchers such as Oaksford (2008) suggest that stimulus equivalence can to some extent explain the origins of reasoning, memory, and language generation. An equivalence class refers to the shared functional properties of items within that class (or category) which can be assumed to be equivalent (Sidman, 1994). For example, fork and knife are within the same category (or class) of cutlery and share the function “to eat with.” Goldstone et al. (2018) accept that the items within a category can be broadly considered an equivalent class. If a functional-analytic approach can be adopted with regard to simple category similarity, then we propose that this approach can also be adopted to our understanding of the emergence of background knowledge.

## The Limited Success of Linguistical Semantics and Sematic Logic of Artificial Intelligence in Accounting for Background Knowledge

The related field of semantics refers to the study of cognitive structures in the brain from a variety of perspectives including cognitive psychology, neuroscience, linguistics, philosophy, and AI (Löbner et al., 2021). There are several branches in semantics. Formal semantics is particularly relevant to the study of background knowledge in categorization, and can offer some useful perspectives. For example, it does not focus directly on cognition, and instead it focuses on natural language, such as the logical relations within sentence structures (e.g., equivalence) which give meaning to the language being expressed (Löbner et al., 2021). As such, one very important observation made in some seminal work of the formal semantics domain (Montague, 1970; Lewis, 1976) is that meaning embedded in some linguistic content is context dependent. Influenced from this early work, most semantic theories now adopt some form of pluralism about linguistic content and its attributed meaning based on this context-dependency (Potts, 2004; Zimmermann, 2012; Ciardelli and Roelofsen, 2017). Context here can relate to the objective and subjective meaning of a given linguistic sentence, epistemic content, intentional content, etc., in order to assign truth-values to linguistic utterances (Löbner et al., 2021).

This context dependency work has also highlighted cross-linguistic, and cross cultural linguistic differences that can alter and shape the meaning of concepts and sentences, which indicates that linguistic meaning is more complex than the words formed in a sentence (Machery et al., 2004; Smith et al., 2018). One of the perhaps most well know theories to have emerged from this work is called the Sapir-Whorf’s linguistic relativity hypothesis which suggests that the structure of language affects the speaker’s cognitive worldview (e.g., Eskimos have many more words to describe and conceptualize snow than Europeans), and that the perception people develop about some concept is relative to the context of their spoken language (Koerner, 1992; Hussein, 2012; O’Neill, 2015).

Another branch of semantics which is relevant to studies in categorization theory of background knowledge is lexical semantics, which emphasizes linguistic concepts in the context of frames which attribute-value structures (Fillmore, 1976; Barsalou, 1992, 2014). Here, a cascade is a combinations of frames in a tree, and category prototypes are structured within these knowledge trees. The frames mediate the input information and output behavior through a Bayesian inference model of category learning (Taylor and Sutton, 2021). Frame-theoretic representations in the form of recursive attribute-value structures organized around a central node is clearly an improvement compared to simple feature list models (Anderson, 1991; Sanborn et al., 2006; Goodman et al., 2008; Shafto et al., 2011). For example, a feature list would simply consist of a list of features, e.g., fur, black, and soft, however, a semantic cascade of frames can add further information to the features, such as representing how these features are related by defining each feature as a value of some attribute. For example, by specifying through a frame that fur has two attributes – color (black) and texture (soft). These types of Bayesian models can allow frames to assign more or less weight to attribute values given their importance in the category structure.

These semantic theories have not just influenced our understanding of cognition, but they have a long standing bidirectional relation with influencing and being influenced by work in computer science, specifically in the area of attempts to formalize approaches of logic and linguistics for general knowledge in the discipline of AI through the development of knowledge tree attribute-value matrices of computational linguistics (Gärdenfors, 2004; Gardenfors, 2014). AI was developed in the 1950, and were largely based on mathematical logic programming such as propositional logic (assigning truth and false to statements), first order logic (formulas which specify some relation to objects) and second order logic (specifying relations between relations), as well as conditional logic (IF-THEN rules) (Luger, 2005; Stuart and Peter, 2020). In line with semantics which seek truth in statements, logical statements of truth can be interpreted as formal semantics, and drew from these early AI approaches. In an example of first order logic, the statement “there is a mother to all children,” can be stated as:


(1) (∀x)(child(x)→(∃y)(mother(y,x)))

Whereby ∀, expresses “for all instances,” child is *x*, → is a connective between the two statements (where this connective is only true if both statements are true), ∃ expresses “there exists,” mother is *y*, *y*, *x*, denotes *y* for instance *x*. This is constructed as a statement of truth, through the use of the connective symbol (i.e., by explicitly stating, when this statement is true, this statement is also true).

These semantic approaches developed further as cognitive semantics emerged in the late 1970s out of cognitive linguistics and mathematical logic, largely based on the initial work of Noam Chomsky’s (a linguist) semantic structures, theory of generative grammars (Chomsky, 1957), and largely due to the dissatisfaction of behavioral approaches (at the time) to explain a model for natural language (Andresen, 1991; Harris, 2010, 2021). Hence, this early work in AI and cognition was heavily influenced by formal approaches of cognitive semantics (Kuznetsov, 2013; Stuart and Peter, 2020).

However, these approaches have been entirely unsatisfactory, and have only led to very narrow approaches to AI, and have not led to the ability of AI to develop broad and deep general knowledge about the use of language in the world (Floridi, 2020; Stuart and Peter, 2020; Toosi et al., 2021). Perhaps the biggest limitation with this approach is that, though context was highlighted early as important, these development were based on narrow and overly simplistic forms of knowledge trees, which do not capture rich contextual structure in the real world. The problem of developing AGI has been suggested (Mitchell, 2021) to depend on some more generalized accounting of (background) knowledge that goes beyond simple statistical, or similarity methods which are limited in nature.

Since this early work on semantics there has been many breakthrough with machine learning approaches, and mathematical models of reinforcement learning (behavioral approaches) are making a return (Silver et al., 2021), and may (with functional context) be an important part the solution for AGI. Deep learning neural networks (DNN) have recently been developed with the advent of powerful processing computers (which as not the case decades ago) which has allowed researchers in the area of semantics to exploit (Rogers and McClelland, 2011) which extends previous work of distributed memory model (McClelland and Rumelhart, 1986) and the semantic memory models (Hinton, 1986; Rumelhart, 1990; Rumelhart and Todd, 1993; Hinton and Anderson, 2014). This allows the model to capture complex structure based attributes as well as relational components, and is perhaps the most promising work within semantics (which is relevant to background knowledge) at this time. This, therefore, may represent a promising way forward for modeling background knowledge in a categorization setting, and general knowledge more widely. However, the relational attributes within this approach are still very limited (e.g., “can,” “is,” etc.), representing simple word structures, and may need to be further developed and updated from a model which specializes in deep contextual (relational) learning.

## A Functional Contextual Account of Background Knowledge – A Potential Solution

One approach which we may look to progress semantic models is Relational Frame Theory (RFT), which is a modern behavioral theory of human language and cognition, which is rooted in functional contextualism (it represents the formal organizational model of functional contextualism), and offers a formal model within a functional-analytic approach to higher cognition (Barnes-Holmes et al., 2001; De Houwer, 2013). In doing so, the theory extends Skinner’s concept of verbal operants to include generativity (thus accounting for Chomsky’s criticism of the behavioral theory) to capture greater complexity within cognition. From an RFT perspective, language can be thought of as learned patterns of generalized contextually controlled, derived relational responding (somewhat similar to inference), called relational frames (Barnes-Holmes et al., 2001). With its basis in functional contextualism, the theory focuses on the role of context (via contextual cues) in facilitating the emergence of specific patterns of relating and how (behavioral) functions come to be attached to these patterns.

Relational responding can be either arbitrarily applicable or non-arbitrary in nature. Non-arbitrary relational responding is based on physical features (such as magnitudes of size, shape, or color) of the stimuli involved (not unlike similarity theories in the categorization literature). By contrast, arbitrarily applicable relational responding is *not* based on formal stimulus properties but is instead largely controlled by historical contextual learning. The theory specifies several different patterns of arbitrarily applicable relational responding, including (but not exclusively): co-ordination (e.g., stimulus X is equivalent to stimulus Y); comparison (e.g., A is bigger than B); opposition (e.g., up is the opposite of down); distinction (e.g., C is not the same as D); hierarchy (e.g., an Alsatian is a type of dog); and perspective-taking (often referred to as *deictic*) which involves the interpersonal (I vs. YOU), spatial (HERE vs. THERE) and temporal relations (NOW vs. THEN).

Relational Frame Theory appears to share some features with rule-based categorization but relies specifically on a history of operant conditioning across a wide range of situations in order for the early patterns of relating to be established. Indeed, the focus of a relational frame, and its definition, require the development of three properties: (1) In *mutual entailment* (ME), relating to one stimulus entails relating to a second stimulus. For example, if A = B, then B = A. Similarly, if you are told that X1 is smaller than X2, you can derive (entail) that X2 must be bigger than X1. (2) In *combinatorial entailment* (CE), relating a first stimulus to a second and relating the second to a third facilitates entailment between the first and the third stimuli. For example, if you are told that A is greater than B and B is greater than C, you will derive (combinatorially entail) that A is greater than C and C is less than A. (3) The third core property of a relational frame is known as the transfer (or transformation) of stimulus function (ToF), through which the functions of any stimulus that participates within a relational frame may be transferred or transformed in line with the relations that stimulus shares with other stimuli also participating in that frame. For example, consider a situation whereby a shock is delivered to your arm each time stimulus A appears on a screen. If you are then told that stimulus B is greater than A, fear in the presence of B can actually be stronger than fear in the presence of A, even though shock was directly paired with A and not B. This is because fear as a behavioral function that is established to A is transferred to B in greater magnitude because of the established relation that B is greater than A. In other words, the fear function is transformed (increased) because of the comparison relation between A and B (Dougher et al., 2007).

**FIGURE 1:**
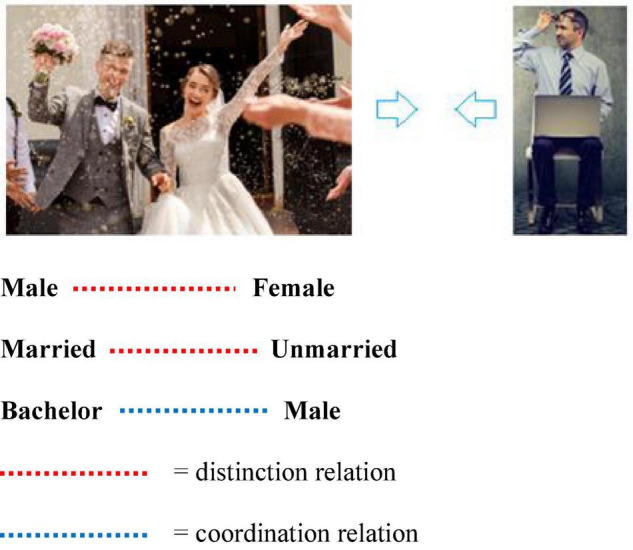
An RFT interpretation of a simple relational frame of coordination between the concept bachelor and the concept male. This includes a distinction relation between male and female and a distinction relation between married and unmarried(Images from adobe stock with license and permission to use and modify given. Credit for image on right “pathdoc” and image on left “wedding photography”).

For RFT, complex social concepts or background knowledge are established as broad patterns of arbitrarily applicable relational responding (AARR) in the form of complex relational networks and adjoining behavioral functions. For example, the accepted social knowledge that “all bachelors are males” involves at least the following: a co-ordination relation between the concept bachelors and the concept male; a distinction relation between male and female; and a distinction relation between married and unmarried (see Figure 1). In other words, for RFT we acquire broad and complex knowledge through verbal operant conditioning, including how males and females differ, why a bachelor is a man, but why a man may not be a bachelor. Each, on the surface, appears to be a simple concept or a comparison of simple concepts, but for RFT the stimuli or concepts participate in much more complex patterns of related or integrated social knowledge. This, therefore, does away with any need to specify a necessary or sufficient feature, and instead prefers context dependent relational networks and associated behavioral functions, which may provide a more accurate account of how causal connections and interrelations within background knowledge about these concepts emerge within their context and influence categorization behavioral decisions about these and other related concepts. As the next section will demonstrate, this approach may help extend existing background knowledge categorization models in interesting ways to help resolve the knowledge selection problem. It will do this by offering some insights into how knowledge is selected in background knowledge categorization tasks, by specifying the context in which stimuli is presented (i.e., via contextual clues) and explain how the relevant functions of stimuli emerge as they dynamically change through inference based (derived relational) networks. Crucially, this will offer some explanation as to how category decisions based on background knowledge are made.

## Specific Examples of Categorization Modeling for Background Knowledge and Why a Functional Contextual Model May Improve on These Accounts

In one attempt to use a similarity-based model to address background knowledge, Heit developed the integration model of categorization (Heit, 1994), which has been explored in a number of background knowledge studies (Heit, 1994, 1995, 1998). In this approach, Heit had modified the similarity based exemplar model (Medin and Schaffer, 1978) in a way which would take into consideration influences of background knowledge such as congruence in category learning (Heit, 1994, 1997).

Several studies have found supporting evidence for congruence in category learning. For example, if a question is presented to participants which is congruent with their existing background knowledge, this will more likely facilitate their memory (Greve et al., 2019), learning of relational properties (Ostreicher et al., 2010) and the classification of new items as consistent with the background knowledge they have (Heit, 1994). For example, in a study presented by Heit (1994), participants were asked a congruent question such as ‘how likely is it that someone with expensive trainers is a jogger?’, and this was compared with an incongruent question such as ‘how likely is it that someone who attends parties is shy?’ It was found that participants were more likely to judge a new person as being a jogger when asked a question which was congruent to the background knowledge of the participant rather than incongruent.

However, in the same study by Heit (1994) it was demonstrated that congruence with background knowledge was not always the strongest factor in influencing categorization behavior. Specifically, Heit found that integration model outperformed the distortion model in predicting whether a group of individuals were “joggers” or not, given a set of categorization behavior. During an experimental setting in this study, participants were shown a training set of joggers (including associated behavioral characteristics). The distortion model predicted that the learned exemplars about these joggers in this experimental setting would undergo a form of distortion in memory to fit better with the previous background knowledge about joggers that the participants had learned over the years (such as joggers typically have expensive trainers), in order to be more congruent with their background knowledge. However, instead of undergoing distortion, the “new joggers” selected by the participants were more similar to the “jogger” training set shown, as opposed to features which were consistent with background knowledge (features typical to joggers).

There are two problems with the above approach. Firstly, and most importantly, congruence in category learning only explains why knowledge is selected in very specific cases and tasks. This is an incomplete model of background knowledge. Secondly, the model fails to predict or explain in what cases some similarity function, or some distortion of memory should be preferred in order to increase congruence with background knowledge. The ability to capture and predict cases of congruence with background knowledge may be captured more accurately utilizing the functional contextual (RFT) model. The RFT approach assumes that relational framing occurs amongst the properties of background knowledge of a typical jogger and some new instance of jogger, which allows for congruence.

So, in this case the non-arbitrary properties would be consistent with a more similarity based situation, whilst arbitrary properties would be consistent with the background knowledge of the jogger. From there, the attributes of the concept “jogger” are related in the network according to the properties of ME, CE, and ToF, where information is derived within the network according to information which can be coordinated, opposed, or where a distinction is made (hence, for example, coordinated concepts would be more congruent that those which are opposed in the network). Hierarchical information can also be held, such as structuring joggers within the network as containers of their personal values such as “healthy living,” “fitness,” etc., through determining the function of their behavior (i.e., why do joggers jog?) which may give more clues about relevant background knowledge of a typical jogger, and the crucial knowledge which needs to be selected dependent on some experimental context. Crucially, RFT can specify how this knowledge is structured within a relational network, and how context of a categorization task (what it asks the participant to categorize exactly) can help determine which knowledge is specifically selected (i.e., the context determines this). For example, if a participant in a study were asked to categorize the personal values of a jogger, then the hierarchical component of the network may be recalled to help the participant decide that jogger’s value “healthy living” and “fitness” based on the participants functional interpretation of why a joggers jog (i.e., RFT determines functions within context are crucial for understanding which knowledge is selected, and thus provides some insight into overcoming the knowledge selection problem).

The RFT model also goes beyond simple congruence by specifying under which conditions equivalence, opposition, mutual entailment, and combinatorial entailment occur more globally, so its ability to specify precisely how congruence can emerge is understood precisely through the models’ ability to predict under what circumstances entailment is generated in the relation network given very specific modeling of historical reinforcing contingences. Some recent evidence has shown that the model was able to accurately predict relational framing of categories in three domains, which included non-arbitrary, arbitrary containment, and arbitrary hierarchical relations (Mulhern et al., 2017). In that study, language and cognition which are relevant to background knowledge were assumed to be patterns of generalized, contextually controlled relational responding. The researchers suggested that this approach offers a more global accounting of knowledge generation and is in contrast with the more localized environment-behavior interactions prediction of congruence formations as is seen in many categorization approaches used (Laurence and Margolis, 1999; Murphy, 2002; Palmer, 2002).

In another example, rule-based approaches of categorization describe how “if” and “then” logical rules are used to define a category (E. E. Trabasso and Bower, 1968; Smith and Sloman, 1994; E. E. Smith et al., 1998), for example, “if” X barks “then” X is a dog. Evidence has shown that in some situations rules do apply (Rouder and Ratcliff, 2006). It has been suggested that rules can be applied to many settings, such as when recognizing that 683 is an odd number (Armstrong et al., 1983) and why raccoons’ offspring look like skunks but are not skunks (Keil, 1989). However, there are some problems within the categorization literature as current categorization models do not have any means to specify specific rules, identify the context in which rules emerge from environmental stimuli, or how they can be organized into complex background knowledge.

There have been some useful rule based models such as the competition between verbal and implicit systems (COVIS) model (Ashby et al., 1998) which suggests that explicit verbal (rules) and procedural (implicit) systems which integrates information at the point of pre-decision, are adopted depending on the context of the situation. Some evidence has suggested that implicit procedural information is integrated when it is difficult to define the rules verbally, but when these can be defined verbally, they usually supersede the procedural system (E. E. Smith et al., 1998). However, others have acknowledged that the exact interplay between rules and procedural systems has yet to be discovered (Milton and Pothos, 2011).

Some more specific rule-based explanations of the effects of background knowledge on categorization have been applied in conceptual acquisition tasks. These tasks were developed in order to identify situations where the background knowledge may facilitate or hinder concept acquisition. In an example of this, Pazzani (1991) used conceptual acquisition tasks involving photos of people performing actions on objects. Each picture showed either an adult or child performing an action on an uninflated balloon (dipping it in water or stretching it) that varied in size and color (it was either large or small, and either yellow or purple).

Pazzani compared two types of categories – a disjunctive category, and under what conditions would each of these emerge. In one experiment the participants were instructed to either learn about a category of balloons that inflate or learn something about an arbitrary category simply labeled Alpha. The assumption made by Pazzani was that participants in the inflate category would be influenced by their background knowledge about what would be needed to inflate the balloons, whereas no such influence from background knowledge would take place.

In their pre-test study, Pazzani found that the action of stretching a balloon would facilitate participants expectation that a balloon would inflate, and that adults would be more successful at inflating the balloon than children. The stimuli used in the experiment were pictures of scenes which differed on four dimensions; (1) adult or child; (2) stretched balloon or balloon dipped in water; (3) yellow or purple balloon; (4) and small or large balloon. For the disjunctive condition, a disjunctive rule defined the inflate category, i.e., that these balloons must be stretched or inflated by an adult. As the pre-test study showed, this should be consistent with the background knowledge that the stretching of a balloon by an adult is more likely to lead to the balloon being inflated (i.e., adults are stronger and more capable to inflating a balloon than children, as well as the knowledge that balloons are stretched when they inflate). In the conjunctive condition, the target category (Alpha) was defined by the arbitrary rule, that these must be small and yellow. This rule is not consistent with any background knowledge about inflating a balloon. As expected, Pazzani found that learning was faster for the disjunctive-inflate condition than the conjunctive-Alpha condition. It was concluded that as the disjunctive-inflate condition was consistent with the existing background knowledge, it was easier to learn than the inconsistent disjunctive-Alpha condition. Pazzani concluded that this was evidence that demonstrated that a simple similarity function in the form of feature selection does not connect category knowledge, rather it is background knowledge which bind the category knowledge in these tasks.

The functional contextual (RFT) approach may be able to improve both the descriptive and predictive power of *why* the disjunctive rule was followed and not a simple similarity function. Here RFT can account and explain the specific context in which background knowledge emerges based on some hierarchical organization of rules which supersede a similarity function. More specifically, RFT can explain the context in *why* the disjunctive rule was followed in the Pazzani (1991) study, as derived relations between predicting whether the balloon would inflate, and the background knowledge provides relational clues about likely hypotheses such as the age of the person carrying out the action, and this (according to RFT) is structured within a complex hierarchical and relational network (i.e., inflating the balloon action “belongs to” a specific person who was stretching the balloon). Similar to the previous example about joggers, the context of the study provides clues as to what the relevant functions of the behavior are (i.e., someone inflating a balloon may be doing so to stretch it), and may, therefore, help determine which knowledge should be selected in the participant’s hierarchal relational network in order to make a category decision. The specification of an explicit rule which is consistent with the hierarchical background knowledge associated with stretching a balloon, may supersede any similarity function in a similar way as any explicit rules supersede implicit integrated knowledge in procedural tasks. This RFT approach, again, may help provide greater context for solving the knowledge selection problem by specifying the functions relevant to a specific context, and outlining how this knowledge is connected dynamically within a participant’s relational network, and utilized in a categorization task that draws on background knowledge.

To support this claim, there has been much empirical evidence for the way RFT structures knowledge within relational networks. For example, evidence of RFT has been able to accurately predict and specify complex ruled based organizations of hierarchical responding in categorization tasks (Greene, 1994; Slattery et al., 2011) which may be seen as a particular type of relational responding called hierarchical relational framing, and which may form via a non-arbitrary relational pattern such as containment. For hierarchical classification, the classes themselves are categorized into higher order classes (Greene, 1994; Slattery et al., 2011). An example of hierarchal classification could be ordering “Alsatian” into (contained within) the category “dog” and “dog” into (contained within) the category “Animal.”

This distinction of hierarchy seems similar to the cognitive interpretation of hierarchy, except the RFT model is providing a broader relational framework in which hierarchical processes occur. For example, in one study of hierarchical relational responding of categories (Gi et al., 2012), there were five phases to the study. In phase one, four arbitrary shaped stimuli were established as, and several contextual cues were given such as “includes,” “belongs to,” “same (similarity).” In the second phase, the arbitrary shaped stimuli were trained and tested for derived arbitrary sameness (equivalence), i.e., between the arbitrary stimuli and some nonsense words. In the third phase, deriving relations of containment between lower (novel and additional) and higher levels (identified through the cues “includes,” and “belongs to”), induced responding in accordance with higher levels in the hierarchical network. In the fourth phase, particular functions were established in particular stimuli (i.e., some stimuli were directly trained to associate a function, e.g., the function of fear) at different levels of the hierarchical network. In the final phase, patterns of ToF were demonstrated where stimuli acquired novel untrained functions because of their position in the network. Therefore, again, RFT can specific complex hierarchical networks for which background knowledge can be stored, relationally structured with other concepts and knowledge, and recalled to help the participant identify a category decision based on clues of functions relating to concepts, events, and behaviors given some specific context, and drawn to make categorization decisions.

Connectionist models in the form of neural networks have also been used to explain the influence of background knowledge (Gluck and Bower, 1988; Shanks, 1991). In these models, category learning is thought to correspond to a set of weighted association of nodes, which activate in response to an input pattern. Choi et al. (1993) used connectionist neural networks to model how learning disjunctively defined concepts is easier than learning conjunctively defined concepts. In connectionist neural networks these hypotheses can be simulated with negative (inhibitory) links between nodes for conjunctions and output nodes which correspond to category labels. After many variations, Choi and colleagues incorporated background knowledge into Kruschke (1992) attention, learning, covering map (ALCOVE) model which represents a hybrid between an exemplar (similarity) and connectionist model. In the original version of a two layer backpropagation model of ALCOVE, this did not fit rule-based data very well, however, when implementing background knowledge biases into and adapted version which consisted of a neural network, whereby the biases were captured by the network weighting (through training), categorization performance improved dramatically. This demonstrates the usefulness and flexibility of neural networks for the study of approximating background knowledge in category learning.

Other notable connectionist methods have included the, and the knowledge resonance model (KRES) (Rehder and Murphy, 2003) and the Baywatch model which is a Bayesian and connectionist model (Heit and Bott, 2000). The KRES model is a connectionist model which takes account of background knowledge, but unlike other approaches, it uses a recurrent network with bidirectional symmetric connections whereby the weights are updated by a Hebbian learning rule instead of a feedforward network which relies on a delta rule or backpropagation. Rehder and Murphy assume that knowledge is directly learned or attributed through an inferential process, and they have shown that some inferential properties can be captured through their network. The Baywatch model is very interesting and has had some success in tackling the knowledge selection problem. This is a neural network model which includes Bayesian probability, and converts the networks activation outputs into a probability measure of the likelihood categorizing in a particular way based on some given background knowledge. In order to do this, the model uses the logistic transformation as outlined in Gluck and Bower (1988) (see formal mathematical approach section below, there, this is utilized and expanded on for an RFT interpretation). The network was able to progressively learn which sources of background knowledge correspond to some target categories – hence identifies in some instances which knowledge is selected in these types of tasks.

However, though these connectionist models are useful, and heading in the right direction, they are currently overly simplistic with a single hidden layer and with only three layers in total. Much of the AI literature suggest that deep (multi-layered) networks are more able to capture knowledge properties (Mitchell, 2021). Furthermore, RFT goes beyond simple inference learning (which the connectionist networks are trying to capture). For example, evidence has shown that by using the RFT model, researchers are able to predict how relationally framed patters of ToF emerged throughout the network, and this makes this framework unique when compared to simple hierarchical or Bayesian inference frameworks of order which are typically described within the categorization literature (Murphy and Lassaline, 1997). The RFT approach, instead, precisely defines the controlling function of the stimuli and under what context does such inference occur. For example, Slattery and Stewart (2014) demonstrated many knowledge based properties which could be captured via the RFT approach, and argued that hierarchical relational framing involves two forms of hierarchical responding, both hierarchical classification as well as hierarchical containment. They found multiple properties of unilateral property induction, transitive class containment, and asymmetrical class containment, which again shows how RFT was able to model complex relational networks within hierarchical organizations applicable to categorization, and beyond the simple inference accounting and knowledge induction of previous connectionist models such as the Baywatch model (Heit and Bott, 2000).

**FIGURE 2:**
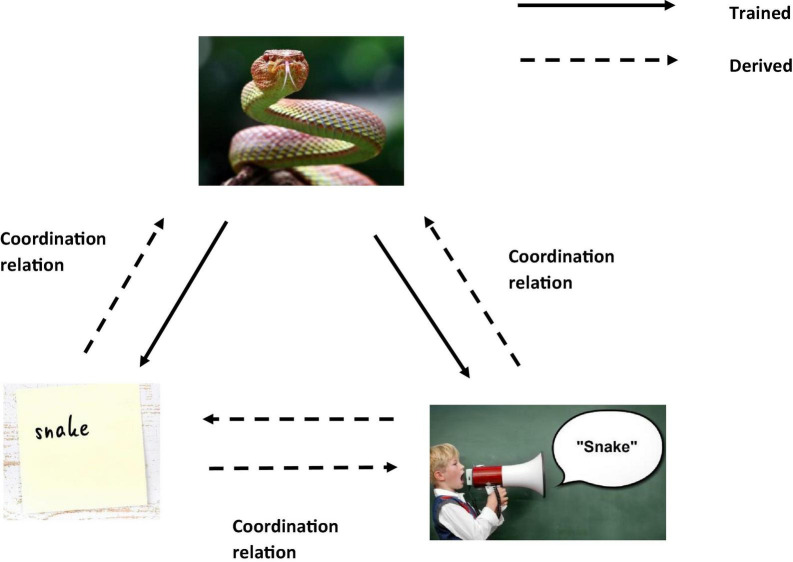
An RFT interpretation of a simple example of trained vs. derived relations(Images from adobe stock with license and permission to use and modify. Credit for image on top “kuritafsheen,” bottom right “Coloures-Pic,” and bottom left “iushakovsky”).

One of the core advantages of RFT’s approach to background knowledge, over and above the previous categorization models mentioned lies not only in its ability to account for high levels of complexity, inference, and context, but also to account for meaning and the effect of a given stimulus, through ToF. Consider the real world example where in a laboratory experiment participants are asked (these would be the dependent measures) to (1) categorize whether the woods are safe, and (2) whether the woods are safe enough to walk through. In this experiment, the participants are told that poisonous snakes live in these woods. The participant when deciding whether the woods are safe or not, may draw on their background knowledge about the snake and themselves. For example, when considering the concept of a snake, the relevant background knowledge can include the common rule “Stay away from snakes, they’re dangerous,” and the very real fear that likely emerges for some when you are near a snake, even when it is in a terrarium. For RFT, fear is an established function of actual snakes and the word “snake” (fear and snake will be coordinated even if you have never seen a real snake). This also applied to the written word “snake” which is also coordinated with the actual “snake” and the verbal word “snake” (see Figure 2).

**FIGURE 3:**
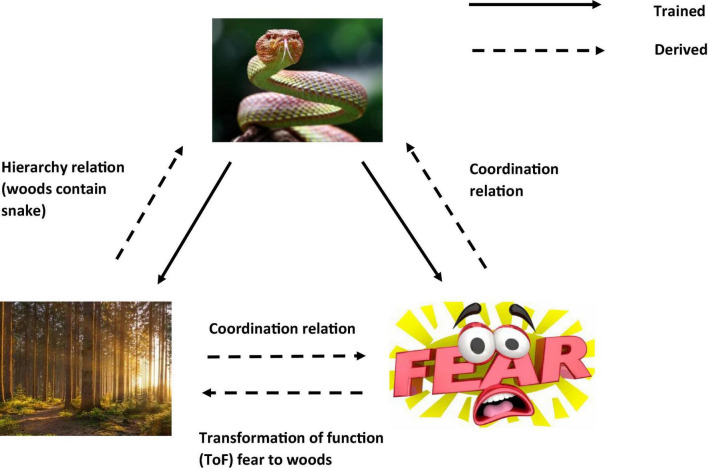
An RFT interpretation of a simple example of transformation of stimulus function, as the individual learns in this context the category “woods are dangerous”(Images from adobe stock with license and permission to use and modify. Credit for image on top: “kuritafsheen,” bottom right: “iQoncept,” bottom left “AA + W”).

So, in a condition when the participant is told that there may be snakes in the woods. Even without seeing a snake there, the function of fear will be transferred to the woods as soon as you hear about the possibility of snakes living in the woods. This is because the concept of snakes (and the attached fear function) is contained (relationally) within the concept of woods, so that the fear of snakes transfers to woods and now you are afraid of just entering the woods (see Figure 3). In other words, woods helplessly evoke fear of snakes. It is even possible that if you are very afraid of snakes, you would avoid woods altogether, without consciously intending to do so (i.e., an avoidance function is established to woods).

As a result, the background knowledge of the participant’s concept for woods as well as their behavior regarding woods can be altered considerably, and RFT provides a model for how background knowledge develops and changes over time. So, it is clear to see through this framework how the background knowledge of snake and woods are derived. In a background knowledge categorization task, where an individual is asked to categorize whether the woods in this circumstance (which contain snakes) is safe, then the knowledge selection clearly involves derived (induced) knowledge of fear functions about snakes and the woods. Of course, though, these derived relations can be scaled up and without any limit on scalability, to include many other derived relations which the individual may network together in terms of the concepts of safe and woods. RFT allows for this continuous scaling up, as new relevant information can be continually added to the relational network model as they are identified, and the framework can thus explain how the processes then further build in additional new background knowledge, and how this will affect a background knowledge related category decision.

It is also important to note that this background knowledge may not have been directly trained and is not based on any form of similarity function between woods and snakes, such as would be stipulated in the exemplar model such as the GCM that try to model background knowledge. In contrast, RFT can explain complex arbitrarily applied relationships among concepts and how these can activate complex behavioral functions in specific contexts. This approach thus goes some way in explaining what types of background information are relevant given the specific learning history of the individual and the context of the categorization task, thus providing greater context for further research to tackle the knowledge selection problem more generally.

In relation to a background knowledge categorization tasks specifically, for example, where we are trying to predict how an individual will categorize whether a particular woods is safe enough to walk through or not, then as suggested, this can be scaled up beyond simple fear of snake. RFT’s framework for describing behavioral outcomes over a wide range of situations, through the frames defined of ME, CE, and ToF, may be helpful as this can help specify complex contextual histories which may help identify how background knowledge is developed, and changes over time, given different contextual settings.

**FIGURE 4:**
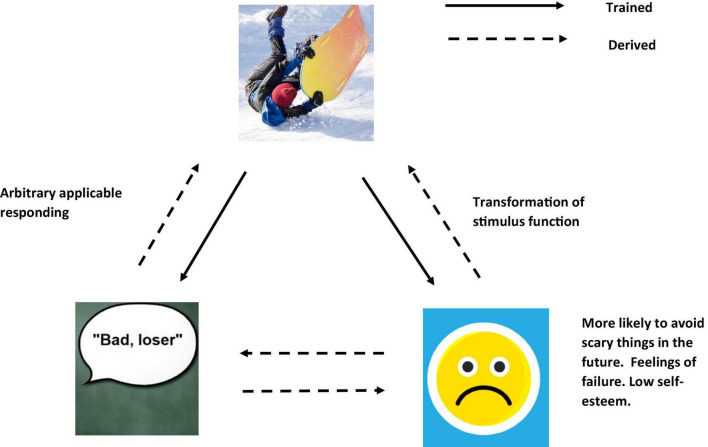
An RFT interpretation of a simple example of transformation of stimulus function, as the individual learns and derives a relation of “self”(Images from adobe stock with license and permission to use and modify. Credit for image on top “Tardigrade,” bottom right “Riska,” and bottom left “Coloures-Pic”).

**FIGURE 5:**
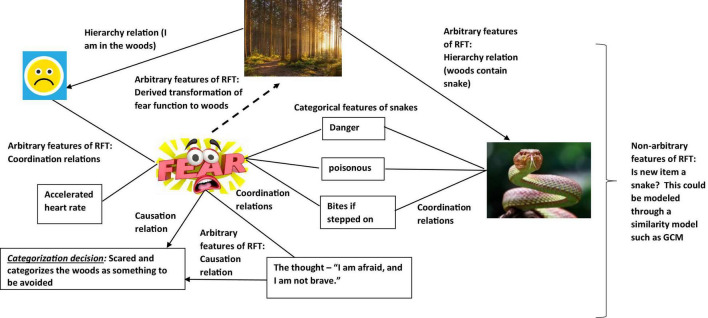
An RFT interpretation illustrating complex frames of derived background knowledge, and how concepts can transfer functions in category learning to develop new functional categories, thus building up the complexity of existing background knowledge(Images from adobe stock with license and permission to use and modify. Credit for image from left clockwise around: “Tardigrade,” “AA + W,” “kuritafsheen,” and “iQoncept”).

In scaling up, further relations can be added. For example, if you had experienced many accidents within your life, and felt that something always bad happens to you, you may frame yourself in the context of “I” as “bad” and “a failure” where “always bad things happen to me,” thus is “likely to get bitten.” This may then have affected your self-esteem and confidence in a negative way (see Figure 4), and with low self-esteem and confidence, with an expectation that something is always bad is going happen to you, this may cause more avoidant type behavior, and lead to greater certainty you would indeed categorize the woods as “something unsafe and to be avoided” (see Figure 5).

**FIGURE 6:**
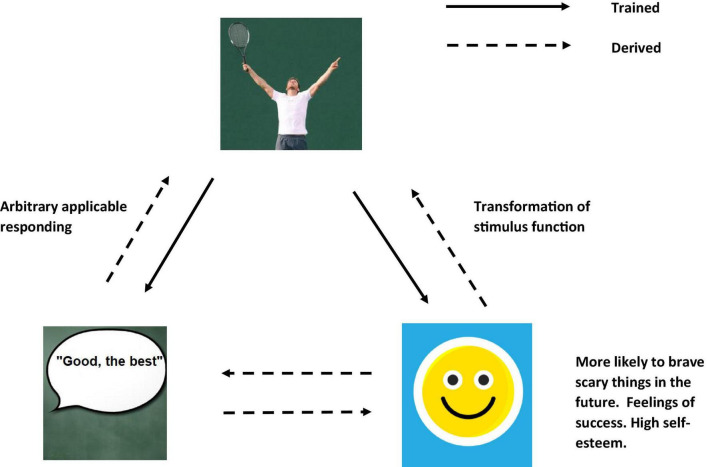
An RFT interpretation of simple example of transformation of stimulus function, as the individual learns and derives relation of “self”(Images from adobe stock with license and permission to use and modify. Credit for image on top “Maridav,” bottom right “Riska,” and bottom left “Coloures-Pic”).

**FIGURE 7:**
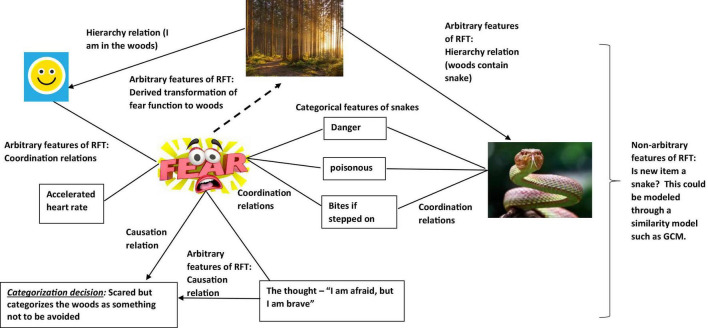
An RFT interpretation illustrating complex frames of derived background knowledge, and how concepts can transfer functions in category learning to develop new functional categories, thus building up the complexity of existing background knowledge(Images from adobe stock with license and permission to use and modify. Credit for image from left clockwise around: “Tardigrade,” “AA + W,” “kuritafsheen,” and “iQoncept”).

Alternately, if the persons contextual history contained many examples of success, and praise from others, they may derive that their derived concept of self “I” is “good” and “successful” where “only good things happen to me” and this may lead to higher levels of self-esteem and confidence (see Figure 6). This may then lead you to conclude that despite feeling fear of the woods, you are confident that you will not get bitten, and as a result may still categorize the woods as “unsafe” but do not categorize the woods as “something to be avoided” (see Figure 7).

## Recent Hyperdimensional Relational Frame Theory Developments Which Expands the Dynamics of Relational Framing Within the Context of Background Knowledge

There have been recent developments of the RFT model worth noting, which maybe additionally useful for studying background knowledge in categorization. The first is the recent development of an RFT framework called multidimensional, multilevel (MDML) framework (Barnes-Holmes et al., 2017, 2020). According to this framework, AARR is assumed explicitly to be able to account for much more complexity than suggested by the standard RFT model (Barnes-Holmes et al., 2001). MDML (RFT) assumes that AARR can develop from not just; (1) mutual entailment; and (2) simple networking involving frames (coordination, distinction, etc.); but also (3) more complex networking involving rules; (4) the relating of relations such as involved in analogical reasoning; and (5) relating relational networks which are involved in extended narratives, and advanced problem solving (which maybe typical for complex background knowledge narratives). The framework also specifies each of these five levels as having multiple dimensions: *coherence*, *complexity*, *derivation*, and *flexibility*, so has a broader analytic framework, which again maybe particularly useful in the study of background knowledge of categorization.

Coherence refers to the extent to which patterns of AARR are consistent with other patterns of AARR. For example, stating “A motorbike is larger than a train” would be lacking coherence with the wider verbal community (who may state the reverse, i.e., “a train is larger than a motorbike”). However, in another context the statement maybe coherent, if for example, the person verbalizing the statement was playing a game, where the objective of the game was to “state the opposite of how you believe concepts actually relate” (Barnes-Holmes et al., 2020). It is perhaps important to note that this extends the notion of coherence as expressed in the categorization literatures (Rosch and Mervis, 1975; Murphy and Medin, 1985) which assumes it relates to a similarly function of intuition, instead MDML (RFT) framework refers to the coherence of more specifically defined (contextual) derived learning.

Complexity refers to the level of detail or density of the AARR. For example, a mutually entailed relation of coordination maybe seen as less complex than a mutually entailed relation of comparison, because the later has two types of relations (if X is faster than Y, then Y must be slower than X) vs. the former which only has one relation (If X is the same as Y, then Y is the same as X). This is likely naturally important for studying background knowledge as some instances of knowledge will be more complex than others and therefore likely to require more modeling efforts to successfully capture such complexity. An important measure here, from a cognitive perspective, could be the level of entropy (uncertainty) in the network, with more complex networks naturally carrying more entropy. The success of the networks could be ultimately assessed by their ability to reduce entropy across the network by accurately capturing environmental patterns of AARR.

Derivation refers to how often a particular derived relational response has been emitted previously. The greater the derivation, the less derived those emitted responses become, because it establishes its own history beyond that of the derived relation that was initially made (the baseline relation). Again, this is an interesting dimension in this framework, and similar patterns of establishment have been observed in cognitive science such as within the unitization of sequence information. After much practice, the sequence information have been found to become compressed into their own unit of information inseparable to that of its sub-components (Perlman et al., 2010, 2016).

Flexibility refers to the extent to which a given instance of AARR is modifiable by current contextual variables. For example, if someone is asked to respond with the wrong answer to the question “Which is larger, a motorbike or a train?”, the easier this would be for the participants to achieve, the more flexible their corresponding AARR network would be. Flexibility maybe particularly important when modeling background knowledge under changing context such as during task switching studies, and maybe heavily related to the other dimensions in this MDML framework such as coherence.

The MDML (RFT) framework, however, has been even further developed, as it focused mainly on entailment relation (*C*_*rel*_) properties of AARR and largely ignored functional (*C*_*func*_) properties of AARR. So, it was further integrated with another RFT adapted framework called differential arbitrary applicable relational responding; DAARRE (Finn et al., 2018) specifically for specifying functional (*C*_*func*_) properties explicitly in the model. This integrated framework (integrating MDML with DAARRE) has been now called the hyperdimensional multilevel (HDML) framework (Barnes-Holmes et al., 2020). The HDML framework for RFT builds on the previous properties of RFT (DARRE and MDML) and specifies the dynamic interplay of AARR (called ROE; relating, orientation, and evoking) in verbally able humans, whereby: (1) *relating* refers to the myriad ways of events maybe verbally related; (2) *orientating* refers to noticing or attending to stimulus events; and (3) *evoking* refers to whether some noticed stimulus (e.g., a concept) or event is functionally appetitive, aversive, or neutral.

This recent work is likely useful in the study of background knowledge of categorization, as background knowledge is likely to involve complex extended narratives, which HDML (RFT) can account for through specifying, for example, the coherence, complexity, derivation, and flexibility of relevant AARR, and dynamically under different context. Specifying relating of relations and relating relational networks maybe particularly relevant, as background knowledge may compromise relating several networks which are related in some way. For example, this maybe applicable to the network for jogger, and a network for city location as in the study example given by Heit (1994), or a network for snake type, a network for the self, and a network for woods in the example provided in Figures 5, 7. This may encourage analysis of the knowledge networks that extend beyond the simple level of the frame and to take into account *C*_*rel*_ and *C*_*func*_ within a broader relational framework.

In an example of complexity, consider the example given in Figures 5, 7. Here, a specification of AARR was given about the individual (verbal) self (“I am… good or bad”) in these scenarios. Here, verbal self becomes a network (see Figures 4, 6) with deictic (perspective-taking) relations (I-You, Here-There, Now-Then) which explain the self as becoming increasingly entangled as the complexity of the AARR network increases (Barnes-Holmes et al., 2020). So, in the example of whether the woods is safe enough to walk through, the verbal self plays an important role in this categorical decision, as well as its associated entangled relating networks. Figures 5, 7 (which are examples of relating relational networks) involve the (network 1) hierarchical *relation* of “I” within the woods and this naturally brings about the relevant AARR network relations that may define the verbal self (“I”) in that context. For example, Figure 4 demonstrates an AARR network relation of an “I” that has derived failure, which then can be explained by HDML (RFT) as the network *orientating* thoughts about failing and getting hurt, and *evoking* feelings of failure, hopeless, low-level esteem, and therefore leads to a greater behavioral tendency for avoidance when confronted by difficult situations (such as a possible dangerous snake in the woods). In contrast, Figure 5 demonstrates an AARR network relation of an “I” that has derived success so *orientates* thoughts about being successful, and *evokes* feelings of being safe and confident, which therefore lead to a greater behavioral tendency for taking greater risk in the face of difficult situations such as when possibly confronted with a dangerous snake in the woods.

These scenarios (Figures 5, 7) relates (relating relational networks) of the verbal self-network 1 with another network (network 2) of the woods, whereby the snake specialist *orientates* the participant toward the danger of the woods, through *relating C*_*rel*_ snake with danger, which *evokes* the feelings of fear and avoidance. This network extends the function (*C*_*func*_) of fear of snakes to fear to the woods (through the relation of woods contains snakes), and relating with the network of self (network 1) which may then ultimately define the outcome of the background knowledge category decision. Therefore, in this framework it is the AARR relating of relational networks which is the correct level of analysis in order to explain the relevant background network knowledge in this case as accounted for by HDML (RFT). Modeling work here could also assess the levels of coherence and derivation there across these networks, and the complexity required to accurately predict categorization decisions involving complex AARR relational networks, as well as the flexibility of the networks under different contextual settings, to further increase the accuracy of the models prediction of some category decision based on some background network knowledge.

Some specific and relevant (to background knowledge) examples of empirical work which support the MDML and HDML frameworks in this area includes rule-governed behavior, which from a traditional behavioral perspective refers to verbal antecedents of stimuli that specify the dependence of relations between stimuli and events (Skinner, 1966). The standard RFT-based operant account (Barnes-Holmes et al., 2001), extends this to involve relations frames of similarity, difference, opposition, coordination, equivalence, temporal, hierarchy, and conditional *if-then* relations, etc. It is perhaps interesting to note that some of these properties are similar to that of logical semantic rules such as the conditional frames. However, some interesting (HDML) recent work has extended this work even further, specifically analyzing the relationship between rule following and coherence. For example, researchers (Bern et al., 2020) have found that coherence significantly impacted upon levels of rule resurgence, and that by manipulating coherence significantly impacted self-report measures relating to certainty in their responding. In other areas of work, participants have shown that they prefer to follow rules which are coherent with the reinforced patterns of relational responding in contrast to rules which are not coherent (Bianchi et al., 2021).

Work on rules and derived relations has also been conducted (Harte et al., 2017), which demonstrated that when participants were either given a direct rule, a derived relation, or no rule, they found that the direct rule led to most rule-persistence, and the rule which contained a derived relation led to more rule persistence than the no rule condition. Another study (Harte et al., 2018) showed that lower levels of derivation generally produced more persistent rule-following than higher levels of derivation. Work has also systematically examined the impact of coherence on persistent rule-following at varying levels of derivation (Harte et al., 2020). The researchers found that by manipulating whether feedback was either present or absent for relevant derived relations when derivation was high influenced the outcomes of rule persistence, contingency sensitivity, and resurgence, as well as (a marginal significance) for rule compliance. They also found that associations between increased rule persistence and increased levels of self-reported compliance were positive.

Ultimately, this recent empirical work is promising and provides and important extension to the RFT analytic level required to study background knowledge for categorization, and to identify an appropriate model for. For example, it applies properties already generally accepted within existing cognitive categorization literature such as equivalence (Oaksford, 2008; Goldstone et al., 2018) but places it within a broader framework for which rules within categories can be studied (e.g., relating rules to other rules, assessing their coherence, and how they are derived, etc.), as well as specifying coherence more broadly, as the framework can draw on analysis of the coherence of any AARR networks which may be involved, and also the complexity, and flexibility of relations specified given some context of the background knowledge information provided. As such, the model has perhaps matured enough to accommodate a comprehensive mathematical framework for the study of background knowledge.

## Some Mathematical Formalization Considerations

In summary of existing mathematical models, similarity (mathematical) models such as GCM and many other exemplar based models have some advantage as they can model non-arbitrary components of similarity based on physical magnitude features (color, shape, size – form), but are limited in that they cannot model arbitrary (rule type) properties. The COVIS model, is designed specifically to capture arbitrary explicit verbal (rules) as well as some procedural (implicit) system components. However, outside of rules and procedural tasks, it cannot explain which knowledge is important to select, which context information is selected in, or how functional properties are determined and can carry through a relational network in a dynamic way. Therefore, in many ways, the COVIS model is limited for the study of background knowledge in categorization and the knowledge selection problem. Models such as ALCOVE, KRES, Baywatch model are neural network models (connectionist methods). These have perhaps the most potential for modeling important constructs in background knowledge which are relevant when making decisions in categorization tasks. ALCOVE (a similarity and connectionist model) was able to model biases in background knowledge. Baywatch is perhaps the most exciting approach in the connectionist categorization as it has made some progresses specifically modeling how background knowledge affect categorization decisions based on a Bayesian probability output of how likely a categorization decision is based on some background knowledge.

Our RFT approach extends the Baywatch model, with some inspiration from recent development of the AI literature which has suggested that deep neural (layered) networks (DNN) are more able to capture deep and rich context within a data set (i.e., the learning history in this case of background knowledge), but structure our DNN in line with a semantic network (Rogers and McClelland, 2011). We utilize the GCM for non-arbitrary similarity matching, expert system based on set theory, as well as deep neural network model to capture arbitrary properties of RFT (i.e., ME, CE, and ToF). This, approach, we believe, demonstrates that RFT can provide a useful and formalized framework to not just model the physical and functional similarities which occur in categories, but precisely how individuals participants engage and select relevant background knowledge in patterns of contextually controlled relational responding which can bring behavior under contextual control (i.e., to determine the category decision), thus, extending work beyond similarity of physical features, as well as simple inference modeling within existing connectionist approaches.

One potential problem with the RFT approach in the context of categorization research, is that a full mathematical model of this approach has not yet be formulated, so it is difficult to directly compare this with other categorization models such as the GCM, ALCOVE, COVIS, KRES, Baywatch, etc., which are all mathematically defined. A formal mathematical account for categorization research may give the model some advantages in terms of setting out very precise and testable predictions in the context of background knowledge. Some researchers (Shull, 1991; Myung and Pitt, 2002) suggest that mathematical models allow for greater precision and succinct predictions about how conceptual terms are related to one another, and provide higher descriptive and predictive power than models which are not defined in mathematical terms. Alternatively, they suggest that models which do not offer some mathematical description can be clumsy for describing the precise conceptual relationships of a model. This is perhaps why in many areas of science such as in medicine, biology, and neuroscience, these have provided such mathematical models in many of their work (Costanza and Sklar, 1985; Kaplan and Craver, 2011; Benzekry et al., 2014). So, as many of the other categorization models mentioned have specified a mathematical description of their model, here, we attempt to ensure consistency with this approach by offering some mathematical considerations when specifically modeling background knowledge effects using RFT. As such, some mathematical description of RFT is provided, for those who are mathematical modelers and who may be interested in these developments.

This section is structured in the following way; (1) demonstrating that the literature on AI indicates that DNNs have made huge advances in deep discovery of properties (such as with the game of Go) and are highly applicable for developing a model of context and background knowledge in which the properties of RFT emerge such as relational frames of coordination, opposition, etc. This will allow for precise and testable predictions to be made in laboratory experiments; (2) non-arbitrary properties of RFT can be modeled through a similarity function of the GCM, whilst the more important (for background knowledge) arbitrary properties of RFT can be modeled through expert systems based on set theory (built from the feature outputs of the DNN); (3) that the DNN learning approaches from the AI literature can be applied to neural networks of Background knowledge such as by extending the Baywatch and semantic network architectures model specifically; (4) that these multi-modal approaches combined offer the most promising position to formalize the RFT model suitable for testing predictions of how background knowledge emerges and affects category decisions such as in a situation where an experimenter were to ask a participant to categorize, for example, whether the woods safe or not given some context.

So, starting with the AI literature, it can perhaps be assumed that any mathematical model for background knowledge may benefit from drawing upon the many resources and recent developments in the field of machine learning (Silver et al., 2017; Brown et al., 2020), as many of these approaches are tackling a similar problem to background knowledge in AGI research, so, they may be of some benefit here. RFT is largely a relational networking of concepts defined through arbitrary language processes, so some of the developments within the AI community toward developing a machine learning approach, such as those suggested for natural language processing (NLP) may be directly applicable here (Greenway et al., 2010; Berkout et al., 2019).

The relevance of natural language is that it often draws upon background information in identifying the meaning and context of words. This is therefore an important area, however, there is an important difference, as the focus here is on background knowledge, and implementation of such knowledge specifically relevant to categorization studies, and not the generation of language (e.g., parsing and token tagging parts of speech into syntax trees, which occupies much of NLP) (Collins and Koo, 2005; Abebe and Tonella, 2010; Dos Santos and Zadrozny, 2014). It is also perhaps important to note that any implementation of an RFT mathematic model within the context of background knowledge, should be considered developed for the purposes of modeling category learning of background knowledge specifically, and not a general model, which may have to be adapted for other studies and context to fit specific purposes (such as NLP).

There have been some interesting advances in the area of machine learning artificial intelligence (AI), worth noting (as some of these approaches will be utilized in our model), such as DeepMind’s AlphaGo (Silver et al., 2017) winning against the highest ranked player in the world at the time of play (Lee Sedol) in the game of Go. This used novel applications of a Monty Carlo tree search algorithm, as well as deep learning and reinforcement learning approaches. Open AI’s GPT3, on the other hand, has made some advances in the area of NLP (Brown et al., 2020) which utilize novel deep learning methods such as the use of a transformer based neural network which is specialized for text classification, and allows it to predict, and create natural sounding text.

Perhaps the most interesting part of Deep Mind and GPT3 and may be key to their success, was their use of modeling with high accuracy very complex and noisy data through the use of deep learning networks. However, GPT3 is not designed to build on any knowledge structures, instead it utilizes vast DNNs (currently 175 billion parameters for GPT3) to scrape (learn from) the internet such as Twitter and Wikipedia, through application programming interfaces (APIs), to understand patterns of text but without any knowledge development policies of the text itself. Hence, this approach does not involve any account of knowledge representation, however, these kinds of deep learning methods could be applied for the purposed of modeling feature representations and associated RFT relational frames (context) within complex and noisy background knowledge information in order to identify what properties are important under what relational context.

Deep learning networks can be thought of as a regression and classification approach, which can pick up non-linearity within the data (Detienne et al., 2003; Lowe et al., 2003, 2017). However, like DeepMind and GPT3, it is unlikely that deep learning alone will be sufficient for background knowledge categorization tasks, as an expert system would need to specify how RFT organizes the knowledge (i.e., ME, CE, and ToF), and specifically how complex relations emerge between relational networks identified by the DNN (as specified by RFTs most recent HDML framework) relating relational networks which seem relevant in the context of background knowledge.

Deep networks are able to identify non-linearity well, but for simple problems they tend to overgeneralize (Rumelhart et al., 1986). So, an expert system can be usefully applied to avoid problems such as the bias-variance dilemma which is defined as the trade-off between data fitting and generalization when dealing with simple vs. complex background knowledge problems (Geman et al., 1992). In this case, here we propose a model which has several policy modules (expert and neural networks) to account for both arbitrary and non-arbitrary components of the RFT model.

It is important to remember that RFT specifies two key types of relational responding, that is the arbitrary and non-arbitrary responding. Arbitrary responding (the most interesting component of the RFT model) relates to relating through relations (and networks of relations) such as equivalence, opposition, distinction, etc. (see section on a functional contextual account of background knowledge), so for example, an object may be 1 by1 meters and another 10 by 10 meters, but they can arbitrarily be labeled as equivalent (despite obvious physical differences in size) given some context. However, non-arbitrary responding relates to a similarity function, i.e., which objects are most similar to each other in terms of physical magnitude (i.e., 1 by 1 meter objects are similar only to other 1 by 1 meter objects). RFT can define both of these properties, and so can our implemented RFT model through the different modules.

So, the GCM similarity model may be well placed for categorizing non-arbitrary physical feature based information of RFT, whilst both unsupervised and supervised deep learning networks may be optimal for learning the non-arbitrary components of relational representation. Hence, the specialized GCM module based on similarity functions and RFT expert module are useful for localized problems whilst the networks learning structure can be used to identify learning context about where ToF, ME, and CE occur, as well as when specific contextual functions play a role in background knowledge. So, the combining expert network components may be optimal, as suggested in previous studies (Ogidan et al., 2018).

Therefore, in our specification of non-arbitrary cases where physical features and dimensions are given, an expert system, in the form of the GCM model for simple similarity-based processing, and within the context of background knowledge, could be usefully applied. This approach is similar to that applied in the attention, learning, covering map (ALCOVE) model (Kruschke, 1992), whereby a feedforward neural network was combined with the GCM specifically for exemplar learning. The specific difference here is that Kruschke only modeled similarity in the form of exemplar theory whilst we were utilizing the GCM for only non-arbitrary situations where similarity exemplar modeling may be useful (arbitrary relational functions are modeled through the neural networks separately).

The GCM assumes that a new exemplar is categorized on the basis of greatest summed similarity. In this way, the similarity of a new item is summed with all of the items in each of the categories and a classification is made into the category with the greatest summed similarity. Thus, a new exemplar will be classified with category A and not category B if it is more similar to A’s exemplars than B’s exemplars. Specifically, a mathematical description of this model utilizes multidimensional space (Euclidean distance and city-block metric) to represent the exemplars. This can be denoted as follows, whereby the decision (or behavioral) probability of making a category A classification when given stimulus *S*_*i*_, when given only two possible categories (A and B), is expressed:


(2) $$P\left(A|X\right)=\frac{\beta_{A}\eta_{XA}}{\beta_{A}\eta_{XA}+\beta_{A}\eta_{XB}}$$

Where, *P* is the probability of making a category *A* response, when given instance (stimulus exemplar) *X*. β_*A *_is a response bias toward category *A*, whilst η_*XA*_ and η_*XB*_ are the similarity measures in the form of summed similarity (with quantifiable magnitudes such as size, color sound, etc.) of stimulus *X* toward all stored (in memory) exemplars of categories *A* and *B*, respectively. The summed similarity, can be given specifically as:


(3) $${\text{η}}_{XA}=\sum_{j\in A}\exp\left\{-c{\left[{\left(\overset{D}{\underset{k=1}{\sum }}w_{k}|y_{xk}-y_{jk}{|}^{r}\right)}^{1/r}\;\right]}^{q}\;\right\}$$

Where, *c* is an overall scaling (sensitivity) parameter, *r* is a Minkowski distance metric parameter, whereby *r* = 1 is a city-block metric, and *r* = 2 results in Euclidean distances. *q* determines the shape of the similarity function, *y*_*xk*_ and *y*_*jk*_ are the coordinates of stimulus *X* and the *j*th stored exemplar on dimension *k*, respectively, and *w*_*k*_ are the dimensional attention weight of dimension *k*.

In order to formulate the arbitrary RFT components, a similarity function such as the GCM is not applicable. Instead, an expert system denoted through set theory can be used mathematically describe the expert non-arbitrary relational constructs (for ME, CE, and ToF). Hence, equivalence from RFT can be stated as follows for equivalent relational properties between two sets using set theory (the three horizontal bars sign denotes equivalence):


(4) ARBx≡ARBy

However, equation 4 can be expressed more succinctly in the form of ME if the symbol | | | is used to denote a shared relation (AND) within the set as suggested in a previous studies (Gilroy, 2015; Edwards, 2021). In the following example of ME, describing a five stripe snake (A) as being “more dangerous” (R_*x*_) than a three stripe snake (B) derives the relation through ME that, therefore, a three stripe snake (B) must be “less dangerous” (R_*y*_) than a five stripe snake (A), whereby a contextual relation is expressed by *C*_*rel*_ within the set. In this way, ME can therefore be denoted as:


(5) C{ARBx|||BRAy}rel

Or in plain English:

In the woods (*C*_rel)_…{a 5 stripe snake (A) is “more dangerous” (R_*x*_) than a 3 stripe snake (B) AND (| | |) a 3 stripe snake (B) is “less dangerous” (R_*y*_) than a 5 stripe snake (A)}.

An additional condition can be included for CE, which can be denoted as:


(6) C{ARBxandBRCy|||ARCpandCRAq}rel

Or in plain English:

In the woods (*C*_*rel*)_….{a 5 stripe snake (A) is “more dangerous” (R_*x*_) than a 3 stripe snake (B) and a 3 stripe snake (B) is “more dangerous” (R_*y*_) than a 2 stripe snake (C) AND (| | |) therefore, a 5 stripe snake (A) is “more dangerous” R_*p*_ than a 2 stripe snake (C) and a 2 stripe snake (C) is “less dangerous” (R_*q*_) than a 5 stripe snake (A)}.

A further condition can be included to account for ToF, whereby^*f*1^ is the function “fear,” can be denoted as:


(7) C[C{ARBxandBRCy{Bandf1RpCBf2Rq|||A}f3}rel]func

Or in plain English:

*C*_func_ – WHEN told dangerous 5 stripe snakes live IN the woods.

C_rel_ – WHILE talking to a snake specialist, and deciding whether to walk through the woods or not.

Here → is used to show the direction of the ToF from one stimuli to another.

Woods (A) is “related to” (R_*x*_) you (B; as you decide to walk through the woods) and you (B) are thus “related to” (R_*y*_) dangerous 5 stripe snakes (C; who you are told live in the woods and may encounter one if you decide to walk through the woods) THEN you (B) are “fearful” (share functional property of fear – ^*f*1^_*Rp*_) of woods (C→A ToF; as you have been told the snakes that live in the woods are dangerous, so the fear of snakes entails with the fear of woods as you become afraid of the woods) AND woods (C→A ToF) is “feared by” (^*f*2^_*Rq*_) you (B; as the ToF is mutually entailed). This implies that the woods (A) through ToF now has the function of fear (^*f*3^), and causes the feeling of fear when you think about walking through it.

In addition to this expert system, this may be supplemented by unsupervised learning neural network, in the form of a self-organizing map (SOM) (Kohonen, 2012) to support the discovery of learning context, such as under what context should (as predicted by the RFT model) mutual entailment, combinatorial entailment, and transfer of functions occur. This approach is an unsupervised clustering method which has already been formulated in the context of relational learning for RFT specifically (Ninness et al., 2005) to identify situations (types of learning problems) where ToF was not learned (i.e., there were errors in a task) after training, and which learning situations ToF occurred. This type of methodology could thus be applied more concretely to the area of background knowledge in categorization, in identifying the types of learning parameters and context for which ToF, ME, or CE, arise in background knowledge (e.g., to identify what learning context allows ToF to emerge as in the example outlined in Figures 5, 7).

A generic version of the SOM (Kohonen, 2001) used in the Ninness et al. study for ordering the mapping into a two-dimensional grid, giving a model *m*_*i*_ whereby data can then be considered *n*-dimensional Euclidean vectors, can be given here. Here, *t* is the index of data items in a given sequence and *ξ* is a given weight. This can be denoted as follows:


(8) $$x\left(t\right)=\left[\xi_{1}\left(t\right),\xi_{2}\left(t\right),\ldots ,\xi_{n}\left(t\right)\right]$$

The model is iteratively updated. The *i*th model is defined as *m*_*i*_(*t*) and the new value *m*_*i*_(*t* + 1) is computed iteratively from the old value of *m*_*i*_(*t*) as new data item *x*_*t*_. The index *i* refers to the model under processing, and *c* refers to the index of the model which has the smallest distance from *x*(*t*) in the Euclidean signal space. α(*t*) is a scalar factor that defines the size of the correction, and its value decreases with the step indexed *t*. The factor *h*_*ci*_ is a smoothing kernel, called neighborhood function. When *i = c* the neighborhood function is equal to 1. Its value decreases when the distance between the models *m*_*i*_ and *m*_*c*_ on the grid increases. This can be denoted as the following:


(9) $$m_{i}\left(t+1\right)=m_{i}\left(t\right)+\alpha \left(t\right)h_{ci}\left(t\right)\left[x\left(t\right)-m_{i}\left(t\right)\right]$$

Then new models are computed as the following, whereby *n*_*j*_ is the number of computed inputs into node *j* and node *j* runs over other nodes in the neighborhood of node *i*. In order to update *m*_*i*_, this scheme is iterated a few times using the same data to determine the mean ${\bar{x}}_{j}$:


(10) $$m_{i}=\sum_{j}n_{j}h_{ji}{\bar{x}}_{j}\sum_{j}n_{j}h_{ji}$$

In addition to the unsupervised SOM approach, deep learning a DNN (as the literature on AGI suggests) can also be utilized which has some unique advantages over SOM in some instances. One problem with SOM is that the mapping topography needs to be specified by the user, and it does not explore deep learning association properties (Golden, 2001). DNNs, on the other hand, can explore these deeper associations in order to identify what relational frames are relevant, and under what context, in situations where background knowledge is utilized in categorization tasks. Deep learning approaches have been used in combination with SOM in other studies to optimize task results (Asghar et al., 2019).

One implementation of deep learning which may be helpful in modeling deep abstract arbitrary features in the form of functional properties, and can extend the SOM, is from the machine learning (AI) literature, called backpropagation neural network (BPN). Previous background models have focused on Bayesian and connectionist (network) model approaches such as by Heit and Bott (2000). However, Heit and Bott employed a shallow network (3 layer network), and it has only been in the last few years (Jeff Dean et al., 2018; Sejnowski, 2018; Buntine, 2020; Jeffrey Dean, 2020) that advances in computational power has allowed for deep learning networks to effectively process the many levels of weight adjustments and gradient decent required for modeling complex and noisy datasets which background knowledge involves.

This massive scalability of deep learning increases its predictive power immensely as shown in thee AI literature, and is one of the main reasons for the successes of OpenAI’s GPT3 NPL program in modeling complex information patterns (Brown et al., 2020), which has increased the size of its deep network to 175 billion parameters and 96 layers (from 1.5 billion parameters in GPT2), as well as DeepMind’s AlphaGo which also has a large scale deep learning network (Silver et al., 2017; Li and Du, 2018).

So, a DNN maybe useful for learning in what situation and context functional control over behavior and decision making occur within background knowledge categorization tasks. In order to implement this DNN, this starts with the summation of inputs *x* = (*x*_0_, …, *x*_*k*_) multiplied by weights *w* = (*w*_0_, …, *w*_*k*_) and adding in a bias value (usually 1):


(11) $$\sum_{i=1}^{n}\left(x_{i}w_{i}\right)+bias$$

The next step is specifying the activation function for each layer. There are various functions which can be chosen, a useful and commonly used non-linear function is the sigmoid function σ:


(12) $$\sigma =\frac{1}{1+e^{-z}}$$

A cost function *C* (usually sum of squared error) needs to then be defined to allow the network to adjust weight and bias. This can be defined as the average *C*, over the cost function *C*_*x*_ for individual training examples, *x*:


(13) $$C=\frac{1}{n}\sum_{x}C_{x}$$

Gradient decent can be computed through partial derivatives ∂*C*/∂*w* and ∂*C*/∂*b* of the cost function *C* with respect to any weight *w* or bias *b*, so that these weights and biases can be adjusted to minimize the cost (error) function. The gradient of the error which is expressed as ∇*C* and decent can be formulated as (and *b* can replace *w* in the case of bias):


(14) $$\nabla C=\left(\frac{\partial C}{\partial w_{0}}\right),\ldots ,\left(\frac{\partial C}{\partial w_{n}}\right)$$

Perhaps most interesting in Heit and Bott (2000) Baywatch neural network model of is the inclusion of Bayesian probability, which converts the networks activation outputs into a probability measure of categorizing in a particular way based on some given background knowledge. In order to do this, the model uses the logistic transformation as outlined in Gluck and Bower (equation 7) (Gluck and Bower, 1988). Standard Bayes rule can be expressed as the following, whereby *P* is the probability of some hypothesis (*H*) being true, given some evidence (*E*), and denoted as:


(15) $$P\left(H|E\right)=\frac{P\left(E|H\right)P\left(H\right)}{P\left(E\right)},$$

In our model, the same Bayesian probability equation as of Gluck and Bower (equation 7) (Gluck and Bower, 1988), can be utilized at the expert output level. If one lets *S*_*k*_ represent one of the possible stimulus patterns, the logistic function can be denoted as the following, whereby *P*(*R*|*S*_*k*_) is the probability of responding in a particular way given *S*_*k*_. *O*_*k*_, denotes the activation in the output node which results from *S*_*k*_ being presented at the input nodes, θ is a positive scaling parameter, and e is the error term (cost) which in learning the network attempts to reduce.


(16) $$P_{k}=P\left(R|S_{k}\right)=\frac{1}{1+e^{-\theta \left(0_{K}\right)}},$$

Given the complexity of background knowledge modeling, this approach of utilizing several modules of supervised, unsupervised, and expert systems seem the most promising approach. However, although there is perhaps universal agreement that the cortex and other areas of the brain processes information through a connectionist system of connected neurons (Levine, 1995; McLeod et al., 1998; Coltheart, 2004; Lin, 2017), one problem with existing (connectionist) neural networks is that they are unlikely biologically plausible in the sense that there is no evidence that the brain uses backpropagation when learning (Crick, 1989; O’reilly and Munakata, 2000; Whittington and Bogacz, 2017), therefore backpropagation may not be the best way to implement the network. More specifically, in a DNN, the change in each synapse is calculated as a global function of activities and weights of many neurons. However, in order to be biologically plausible (closer to how real neurons signal), the network must perform its learning algorithm locally, and the change in each synaptic weights must entirely depend on just the activity of pre and post-synaptic neurons (O’reilly and Munakata, 2000; O’Reilly et al., 2016; Whittington and Bogacz, 2017).

As a result of this, a large amount of effort has been made into applying more biologically plausible connectionist DNNs, and these have been in the form of Hebbian learning networks which have been shown to approximate the backpropagation learning algorithm, whereby the local synaptic weights depend on the pre and post synaptic activity (O’reilly and Munakata, 2000; O’Reilly et al., 2016; Whittington and Bogacz, 2017). It has been suggested that these biologically plausible DNNs should ensure the following (O’reilly and Munakata, 2000; Whittington and Bogacz, 2017): (1) Local computation, whereby a neuron performs computation on the input it receives from other neurons and weights by the strengths of these local synaptic connections; (2) Local plasticity, synaptic weight changes is dependent on the neurons the synapse connects with; (3) Minimal external control, whereby computation of neurons is performed autonomously with little external control; (4) plausible architecture, where the connectivity patterns in the network should be consistent with the basic constraints of connectivity in the neocortex.

One very interesting adaptation of this comes in the form of predictive coding, from some of the most prominent and widely accepted models of neuron functioning proposed by Karl Friston and colleagues (Rao and Ballard, 1999; Friston, 2003, 2005) and is related to the autoencoder framework (Ackley et al., 1985; Hinton and McClelland, 1988; Dayan et al., 1995), which O’Reilly and colleagues’ Hebbian learning GeneRec algorithm was also based upon, utilized in their Leabra DNN architecture (O’Reilly, 1996; O’Reilly et al., 2016) as well as other autoencoder versions implemented in a biological plausible DNN (Bengio, 2014; Bengio et al., 2015).

Here the backpropagation algorithm can be approximated with the predictive error term in a biologically plausible way, whereby instead of computing the backpropagation gradients via a chain rule which is used in the form of derivatives from calculus in the typical backpropagation network which shows how much the *v*_*i*_ (which denotes the vector of activations in a layer) need to change in order to minimax the cost function *C*:


(17) $$\frac{\partial C}{\partial v_{0}}=\frac{\partial C}{\partial v_{1}}\frac{\partial v_{1}}{\partial v_{0}}$$

Instead, a biologically plausible predictive coding (Hebbian learning) algorithm is utilized:


(18) $$v_{0}=-\in_{0}+\in_{1}\frac{\partial v_{1}}{v_{o}}$$

Where, in predictive coding, ∈_*i*_ is the prediction errors (errors made by parent nodes about the activation prediction of locally connected child nodes). Here, predictions and prediction errors are updated in parallel with only local information. Also, see Whittington and Bogacz (2017); Millidge et al. (2020) for full details. Prediction errors are computed in the following way:


(19) $$v_{b}^{\left(a\right)}=\;{\text{ε}}_{b}^{\left(a\right)}+\sum_{i=1}^{n^{\left(a-1\right)}}{\text{ε}}_{i}^{\left(a-1\right)}{\text{θ}}_{i,b}^{\left(a\right)}\;\;{ƒ}^{\prime }\left(v_{b}^{\left(a\right)}\right)\;$$

Here, prediction errors $\in_{i}^{\left(l\right)}$are computed from the excitation activity of corresponding nodes $v_{i}^{\left(l\right)}$ and the inhibition of the nodes on the next (a higher) layer $v_{j}^{\left(l+1\right)}$ weighted by the strength of the synaptic connection $\theta_{i,j}^{\left(l+1\right)}$. The nodes $v_{i}^{\left(l\right)}$ themselves also make predictions, but on the prediction error from the corresponding level and the lower level which are weighted by the synaptic weights. $f^{\prime }\left(v_{b}^{\left(a\right)}\right)$ refers to the non-linear transformation function which transforms and scales incoming input from lower-level nodes to a variable node. Once the network has reached its steady state it then updates its parameter weights $\theta_{i,j}^{\left(l\right)}$ in this locally Hebbian driven learning which captures synaptic plasticity of real neurons.

## Deep Learning Neural Network Semantic Architecture With Representation and Relational Layers

**FIGURE 8:**
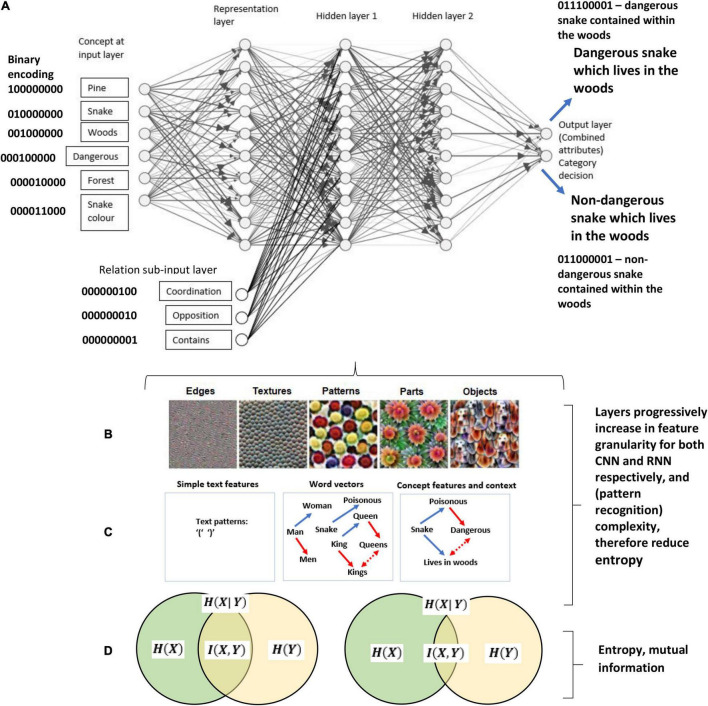
Illustrates RNN encodings for each layer – a representation layer, and a relation sub-input layer for determining output category classifications utilizing relational fames which represent context. For section B (Edges, Textures, Patters, Parts, and Objects), these were taken from Chris Olah (Google Brain Team), Alexander Mordvintsev (Google Research), Ludwig Schubert (Google Brain Team) (2017) (CC-BY 4.0, with permission) https://distill.pub/2017/feature-visualization/. **(A)** represents the neural network encoding relation information; **(B)** illustrates progress layer feature granularity in a typical CNN; **(C)** illustrates progressive contextualized word patterns which detect context in language; **(D)** illustrates decreased mutual information between input and output as the network places focus on important information whilst discarding irrelevant information.

The DNN we use here (see Figure 8A), is similar to the structure of a semantic network (Rogers and McClelland, 2011) which extends previous work of distributed memory model (McClelland and Rumelhart, 1986) and the semantic memory models (Hinton, 1986; Rumelhart, 1990; Rumelhart and Todd, 1993; Hinton and Anderson, 2014). This is optimized for processing information which involve both context independent and context (relational frame) dependent aspects and has been specifically designed for complex contextual knowledge representation which is suitable for emulating basic RFT properties.

This network structure is a parallel distributed processing approach to cognition (McClelland et al., 1986), and assumes cognitive (or behavioral) phenomena arises from the propagation of action amongst connected neuron-like processing units. These neuron unit nodes explicitly encode the state of the environment via direct sensory inputs. The hidden layer nodes mediate the flow of activation encodings between the input and output, and the output nodes explicitly encode representations of potential responses. The propagation of activation is constrained by weighted synaptic-like connections between neuron nodes, and the internal environmental representation take the form of distributed patterns of activations across some subset of hidden units which update overtime in response to input data from the environment.

The function of the semantic network architecture is to generate context and item appropriate inferences about the properties of the inputted data (concepts). It contains representation (concept) inputs as well as relation inputs (see Figure 8A), whereby the relation inputs provide information about the context that influences the representation inputs in the hidden layers (Rogers and McClelland, 2011) as well as similarity structure across relational contexts (Rogers and McClelland, 2008). The input layer directly encodes localist environmental representations of individual concepts, whilst the relation layer encodes localist environmental representations of different relational contexts relevant to the inputted corpus. In the example given the relation al frames are coordination, opposition, containment, but these can be extended to include all of the relational frames within RFT, and the network could learn through a larger training corpus that sentences within a corpus such as “lives in” involves the containment and coordination relations. So, the network develops its own equivalence class for word vectors in order to correctly encode and active the correct relational frames within the relation layer. Crucially, the representation layer encodes a context independent internal representation whilst the hidden layers encodes (receiving input from the relation layer) context-dependent representations.

In this framework, the internal representations that govern how knowledge generalizes are not considered discrete category representations, but instead are patterns of activations across continuously valued nodes distributed across the representation and hidden layers. The patterns of activity can be considered a point in a continuous high dimensional space, which each node encoding one dimension. Unlike exemplar theories in categorization, here, the dimensions of the space do not correspond to interpretable semantic features and there is no storage of exemplars – hence the information is only interpretable when considering patterns of activity across nodes within separate layers. Categories can be output form this model but are not contained within it, and instead correspond to densely occupied regions of the representation (nodes within layers) space.

This approach has the advantage of not needing to specify which categories are stored within background knowledge hence gets around the knowledge selection problem, instead the semantic network derived a function between the input and output properties through its distributed activations across layers. Further to this, it is important to note that this model could be expanded further (as an additional module) to include a reinforcement learning agent structured through a Markov decision process (MDP) as specified in previous work (Edwards, 2021), when more complex decision making is needed which requires the extracting of background knowledge for category decision making. This specifies the probability *P* given some action *a*, and is denoted as *P*_*a*_(*s*, *s*′)^1^, *s* is the current state of the environment and *s*′ is some new state transition if action *a* is carried out. This would help specify which concepts and instances are being reinforced and under which context as knowledge develops into more complex networks.

## Encoding of Information to Network Layers and Graph Visual Embeddings

As with the semantic network of previous work (Hinton, 1986; McClelland and Rumelhart, 1986; Rumelhart, 1990; Rumelhart and Todd, 1993; Rogers and McClelland, 2011; Hinton and Anderson, 2014), our proposed network (see Figure 8A) will store binary encodings to represent the concepts contained within the inputted corpus (within a representation layer), as well as the relational frames (of the RFT model in the relational layer), which will ultimately represent the background information needed to make some category decision (such as whether it is safe to walk through the woods). The output of this network, therefore, will be some category which is relevant in order to make some category decision (the semantic network has been adapted for this purpose).

In terms of granularity of encoding, it is anticipated that like convulsion neural network and other network representation layers (see Figures 8B,C) the level of detail (granularity) increases layer by layer within the DNN (such that layer one captures just the most basic features – edges for images or word structures for a text corpus, layer two captures textures or word relations, etc.), and therefore learning occurs layer to layer, and that each layer has all the information it needs to predict target output plus some noise, whereby noise decreases as the number of layers increase. Figure 8A shows that at the third layer the relational frame (from the RFT model) properties are integrated with the representation properties to forms relational representations (e.g., snake lives in the woods).

From this perspective, every layer becomes a partition of information, and these are known within the information theory literature as successive refinement of relevant information (Equitz and Cover, 1991; Gregor et al., 2015; Liu Y. et al., 2018). So, the input is slowly being encoded and decoded into the target output. From information theory, this can be expressed as the amount of information or entropy (uncertainty or noise) is contained at each layer (see Figure 8D; Tishby and Zaslavsky, 2015; Tax et al., 2017; Achille and Soatto, 2018; Yu et al., 2020). In other words, how much entropy *H* is removed from *X* (input information) at each layer if *Y* (output, respective categories) is known. Here, *H*(*X*) denotes the entropy of *X*, *H*(*X*|*Y*) is the conditional entropy of *X* given *Y*, and *I*(*X*, *Y*) = *H*(*X*)−*H*(*X*|*Y*) denotes the mutual information (between *X* and *Y*). The mutual information between *X* and *Y* should decrease as the important and relevant information is selected, and the noise (non-relevant information) is discarded. This can be represented as a Markov Chain whereby every hidden layer becomes a single variable *h*, in a Markov Chain, represented as *h*_1_, *h*_2_, etc. As each variable in a Markov chain is only dependent on the previous layer, each layer can be observed as its own partition of information.

**FIGURE 9:**
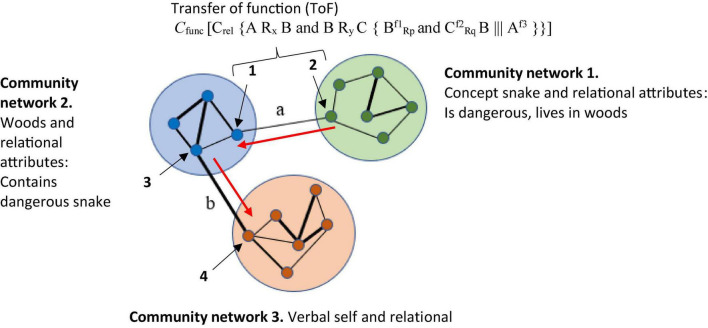
A network structure shows how three community clusters (relating relational networks – of the HDML framework) in a network graph can emerge whereby each cluster has related concepts and are connected (related) to other clusters in the graph where properties such as a ToF from one cluster can influence other cluster networks. Each node represents a unique concept and are connected together in a strong or weak way (depicted by the width of the connecting lines) to other nodes within a cluster. The community clusters (blue, green, and orange) containing the connected nodes are related to one another through some properties of RFT, in this case a transfer of stimulus function. The red arrow indicates the transfer of function “fear” from green community (snake category), to the blue community (woods category), and finally to the orange community (verbal self).

There are several ways to determine and interpret the specific encodings made on neurons within layers and plot them (for visualization purposes) in graphical form (see Figure 9). Coming out of the graph theory literature, explanatory graph analysis (Golino and Epskamp, 2017; Golino, Shi et al., 2020) has been recently introduced. These types of approaches have been useful in estimating a network and then developing clusters of communities of variables from the relationships between the variables within the network (Golino and Epskamp, 2017). This has been particularly useful in psychology in regards to applications of this framework for modeling network psychometrics (Epskamp et al., 2016), through the development of the network graph which are based on identifying the strength of correlations between psychometric variables (Christodoulou et al., 2019; Baker and Berghoff, 2021).

Despite the advantages of the network analysis approaches of direct correlations between psychometric variables, DNNs have the advantage over other approaches of network analysis. This is because they can leverage large amounts of complex data to identify some underlying function that describes some pattern in the data with high dimensionality such as speech, visual, and natural language processing (Liu B. et al., 2017; Deng and Liu, 2018; Voulodimos et al., 2018; Young et al., 2018; Nassif et al., 2019) which is particularly relevant to background knowledge categorization tasks, and emulates the way our brain actually process information (via a Hebbian learning error driven neuronal network) (O’Reilly, 1996; O’reilly and Munakata, 2000; O’Reilly et al., 2016; Whittington and Bogacz, 2017).

This DNN approach has been inspired by recent developments in neuroscience of brain network analysis methods (Liu J. et al., 2017; Garcia et al., 2018), which have shown that analyzing brain neural connections can give important insights into the architecture, development, and evolution of the brain networks. Applied into a DNN framework, here, instead of identifying the direct correlations between psychometric variables (Christodoulou et al., 2019; Baker and Berghoff, 2021), allows for a graph to develop directly from the neural network weight distributions, whereby correlations would be calculated between neuron nodes in order to identify communities within the network (Horta et al., 2021). As weight distributions within a network are difficult to visualize and interpret, Graphs can help the network distributions to become more comprehensible to humans who can then visualize the encodings within the network, allowing for the classic black box problem of interpretability to be overcome.

This problem of interpretability and explainability of how or why the DDN has made some set of connections is not trivial, and makes it difficult for researchers to traditionally understand what is being encoded on each neuron in each layer of the DNN (Towell and Shavlik, 1993; Liu X. et al., 2018; Kumar et al., 2020; Erasmus et al., 2021). This ability to understand some of the encodings can be important with applications such as medical decision making, law enforcement, financial analysis (Horta et al., 2021) as well as when attempting to model and explain the cognitive system (Cichy and Kaiser, 2019; Oita, 2019; Monte-Serrat and Cattani, 2021) such as in tasks relating to background knowledge which my further help researchers understand distributed knowledge encodings across layers and what the weight distributions actually mean in graphical form.

For many years, there been several attempts to extract meaningful encodings from what is traditionally understood as a black box of the DNN connectionist layers (Towell and Shavlik, 1993; Bartlett, 1994; Mak and Blanning, 1998), however it has only been with recent progress in computational resources has this been possible (Horta et al., 2021). Several approaches have made considerable progress in solving interpretability problem (Gilpin et al., 2018) by extracting knowledge from the DNN, such as the pedagogical rule extraction method (de Fortuny and Martens, 2015), and more recently architecture agnostic approaches which do not depend on fully connected networks (Horta et al., 2021).

These recent developments are inspired by co-activation graph methods (Horta and Mileo, 2019) which are similar to functional graphs based on statistical correlations between nodes in the DNN. Within a DNN, nodes represent neurons and the weighted relations between nodes indicate a statistical correlation between activation values. This allows for assessing connection pairs between any layer (including hidden layers) of the network and the output layer. Unlike in previous approaches, this makes it possible to study the relations between neurons within a DNN, whereby knowledge encoded on the in the co-activation graph reflect the knowledge acquired by the DNN in its learning phase (and the stages of evolution of this learning, encoded by the layers). This, therefore, allows for precise measures of encodings across the network (Horta and Mileo, 2019; Horta et al., 2021).

A co-activation graph can be represented (see Figure 9; Horta et al., 2021) through an undirected graph *G* = (*V*, *E*) where *V* = {*v*_0,_*v*_1,…,_*v*_*n*,_} is a set of nodes that represent the neurons of a DNN and *E* is a set of weighted relationships (expressed as edges in the graph) *e*_*ij*_ = (*v*_*i*,_*v*_*j*,_*w*) between pairs of neurons *v*_*i*_ and *v*_*j*_ with weights *w*_*ij*_ are obtained by applying a statistical correlation on *A*(*v*_*i*,_*S*) and *A*(*v*_*j*,_*S*) as depicted in the equation below:


(20) $$w_{ij}=Spearman\_corr\left(A\left(v_{i,}S\right),A\left(v_{j,}S\right)\right).$$

Spearman coefficient is chosen as linear relations are not expected between the neuron’s activations values. Edge weights vary from −1 to 1, and there are three steps on how to develop a co-variation graph. For example, consider a DNN with neurons *n* and a data sample *S* = {*s*_0_, *s*_1_, …, *s*_*n*_}. The three steps are as follows:

The first step is to extract activation values. Here, the DNN needs to be input *S*, then for each neuron *v*_*i*,_ and each data input *s*_*h*_ ∈ S, where 0 ≤ h < m, a single activation neuron *a*_*ih*,_ needs to be extracted. The result is a set {*A*(*v*_0_, *S*), *A*(*v*_1_, *S*), …, *A*(*v*_*n*_, *S*)} where *A*(*v*_*i*_, *S*) represents for the whole dataset *S*, all activation values of each neuron in the network.

The second step is to define and calculate edge weights, which requires defining the relationships between pairs of neurons. For each pair of neurons *v*_*i*_ and *v*_*j*_ the correlation equation 20 is applied and utilizes activations *A*(*v*_*i*_, *S*) and *A*(*v*_*j*_, *S*) in order to establish the statistical correlation for the relationship weights *w*_*ij*_ between each pair of neurons. This allows for a mathematical matrix which contains *w*_*ij*_ for every neuron pair *v*_*i*_ and *v*_*j*_ which can then be utilized to construct a set of edges *E*within a graph and between neuron nodes, and for every neuron pair in the network in order to develop a complete graph.

The third step is to build and analyze the co-activation graph. In order to visualize the graph being developed this needs to be constructed within a computational tool for graphing structures such as Neo4j^2^. This provides a graph, whereby nodes represent neurons at any layer within the DNN that the research wishes to analyze the encodings from, and the weighted edges represents the non-linear correlations between the neuron node activation values.

The final part this approach is the analysis method adopted. Here, we first need to demonstrate that the co-activation graph is encoding the same knowledge as the DNN. This approach has been successfully tested with DNNs, whereby a community structure analysis and centrality analysis are conducted over several data sets (Horta and Mileo, 2019), which helps to observe how a graph algorithm applied to the co-activation graph can explain the DNN model. The community analysis applied to a deeper model helps to establish whether the results are consistent with more complex environments and therefore DNN models. The centrality analysis is applied to study and understand the association between node centrality and neurons which are important in the DNN (Horta and Mileo, 2019; Horta et al., 2021).

The community structure analysis is also important in order to identify interesting properties of the graph and the knowledge held locally with each community. The Louvain community detection algorithm (Blondel et al., 2008) has been applied effectively in previous work (Horta and Mileo, 2019; Horta et al., 2021), and it is useful as it can output a modularity coefficient which indicates how different the community structures identified differ from random graphs. It includes a parameter which allows for the resolution to be adjusted, when seeking larger or smaller communities. Horta et al. (2021) also explored the similarities within the communities and identified that the Louvain algorithm was able to cluster information within the communities which were semantically similar. For example, they found that community one included animals (deer, dog, horse, frog, bird, and cat) in one community whilst in another community this included means of travel (airplane, ship, truck, and automobile). They also found at a higher resolution, the algorithm was able to detect hierarchical class information such as clothing in community one (T-shirt, pullover, shirt, and dress) and footwear in community two (sandal, sneaker, and ankle boot).

Centrality analysis plays an important role in graph analysis, whereby identifying the centrality of a node provides valuable insights of the importance of the neuron node in a graph (and corresponding DNN). There are two different centralities used in this analysis, which are degree centrality and PageRank centrality (Page et al., 1999). Degree centrality of some node *n*_*i*_ presents the number of relationships *n*_*i*_ has with other neural nodes. This can be calculated by simply summing of the weights in the edges that connect *n*_*i*_ with other nodes as in the equation below:


(21) $$D\left(n_{i}\right)=\sum_{j=0}^{N-1}A_{ij}$$

Here, *A* is an adjacent mathematical matrix of size *N*, where *A*_*ij*_,0≤i < N and 0≤j < N. The PageRank expands this equation, one step further by highlighting the importance of a given node’s neighbors, and not just its centrality. Each node’s PageRank is initialized to the value of 1 and then iteratively updated through equation 22, as stated below:


(22) $$PR\left(n_{i}\right)=\frac{1-d}{N}+d\sum_{n_{j}\in S_{n_{i}}}A_{ij}\;\frac{PR\left(n_{i}\right)}{D\left(n_{j}\right)}$$

Here, the total nodes is denoted by *N*, whilst *S*_*n_i_*_ is a set containing the neighboring nodes of *n*_*i*_, *A*_*ij*_, like in equation 21, denotes the weighted edge between nodes *n*_*i*_ and *n*_*j*_. *d* is a damping factor which controls how often random jumps are made to other nodes, whilst *D*(*n*_*i*_) is the degree centrality as in equation 21.

From this, a graph (see Figure 9) is generated from the weight distributions, whereby each node represents a unique concept and are connected together in a strong or weak way (depicted by the width of the connecting lines) to other nodes within a cluster (broadly forming an equivalence class). In the figure, the clusters (blue, green, and orange) containing the connected nodes are related to one another through some properties of RFT, in this case a transfer of stimulus function. Th red arrow indicates the transfer of function “fear” from the green community (snake category) to the blue community (woods category), then finally to the orange community (verbal self). Here, in the example we gave in Figures 5, 7, community network 1 shows the relational attributes of the concept dangerous snakes living in (contained) in the woods, community network 2 shows the relational attributes of the woods also containing dangerous snakes, and network 3 which is the relational attribute of the verbal self. This impacts the decision as to whether the woods are ultimately categorized as safe enough to walk through or not, and is largely based on whether the verbal self has positive relational components (confident and successful), as depicted in Figure 6 or negative relational components (feeling of failing and low self-esteem), as depicted in Figure 4. As each community network is a relational network and are connected to other relational community networks (such as community network 1 and 2) through ToF in this example, this represents relating relational networks as explained by the HDML RFT framework.

It is perhaps important to note that by avoiding the woods, this may further strengthen the individual’s feelings of failure and low self-esteem so may have important consequences in processed based therapy (PBT) work (Hayes and Hofmann, 2017, 2018; Hofmann and Hayes, 2019), and could be applied a clinical analysis and diagnostic tool.

As can be seen in Figure 9, the strength of the connection (edge) *a* is between nodes 1 and 2 and is weak to moderate in strength, so in this case the transfer of function may also be considered weak to moderate in strength and influence over the network. In contrast, the connection (edge) *b* is more bold and this represents a strong connection and increased influence over the network. This means that the verbal self would have a strong influence over the decision whether to decide to walk through the woods, and it also suggests that in the case of an avoidance response, then the negative impact on the verbal self would be strong. In a PBT setting, these techniques could be utilized in a way which brings about relating relational networks in a clinical graph (or structural equation) type approach helping the clinician or researcher to visualize the strength of these relations and therefore judge how likely one relational network would influence another (and hence where to target the intervention). This, therefore, could expand the toolbox of PBT diagnosis on what has already been proposed (Hofmann et al., 2021).

**FIGURE 10:**
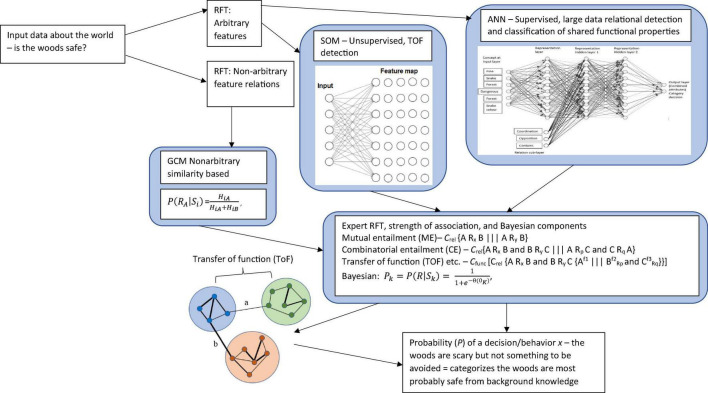
A simple schematic illustration of a multi-level computational model, which identifies GCM similarity in non-arbitrary situations and RFT relations through both unsupervised and supervised network components for learning the RFT framework.

Finally, based on the network and graph weighted outputs, the probably of some decision such as whether the woods is safe to walk through can be made. This relies on an aggregation of the data from the networks in graphical form (to visualize). The expert system then finally can connect community graph networks within the sets of ToF, mutual entailment, or combinatorial entailment as a form of relating relational networks as described in the HDML (RFT) framework, which may be easier than connecting two or more DNNs given the complexity of this task. The complete model with all modules explained in this section can be summarized as depicted in Figure 10.

## Some Novel Experimental Considerations

With the machine learning approaches suggested, it is perhaps important to note that novel experimental methods can be developed which expand on that utilized by Ninness et al. (2005). Ninness et al. identified situations (types of problems) where ToF was and was not learned after training (i.e., by clustering specific errors in learning ToF with specific learning tasks and context).

Similar approaches for deep learning could be developed, for example, which utilize participant confidence scales as inputs within a backpropagation network to represent individuals’ background knowledge (e.g., confidence that the typical jogger in the United States is “healthy,” “runs once a week,” “is honest,” “owns running trainer,” “has two legs,” etc.) and this could be trained on a target of actual jogger reports on how confident they are they have these same features or factual reports about joggers (e.g., 70% of joggers in the United States are healthy, whilst 30% are obese).

Such an approach would allow for the identification of relevant perceived attributes and functions of the target category (e.g., jogger and woods) for specific participant subgroups. For example, given a background knowledge feature list, clustering methods such as k-means on the upper hidden layer could represent a novel way to explore which of the background features given by a given population subgroup are assumed to be important about a concept (e.g., what United States participants believe about joggers in the United States vs. what European participants believe about joggers in the United States), and how this relates to actual properties that “joggers” believe about themselves or other facts known. This type of approach has been used in other areas such as identifying important subgroup predictors in health psychology and other areas (Kimoto et al., 1990; Lowe et al., 2003, 2017).

For this to work, data is converted into *z* scores a two-stage cluster analysis is performed (Milligan, 1980; Clatworthy et al., 2007) which involves a Ward (squared Euclidean distance) to identify the number of clusters (i.e., through the agglomeration schedule and dendrogram), then data conversion into cluster centroids (middle of cluster) followed by a K-means cluster method. This allows subgroups partitions to be identified which contain the relevant background knowledge features and functions. The final step allows the experiment to then compare each feature variable in each group “x” against the mean for that variable across all groups through a comparison of effect sizes calculated from the mean scores and pooled standard deviation.

## Conclusion

The examples above illustrate how RFT offers a compelling explanation of how complex background knowledge emerges, with contextual sensitivities, without relying upon simple rules or formal similarly, as is typically the case with categorization models.

In relation to broader philosophical considerations, cognitive psychology typically takes a perspective of mentalism, such as in categorization research Margolis and Laurence (1999) assume a concept is a mental representation. However, there has been some accepting of functional aspects such as the functional equivalence of Sidman (1994) in the cognitive community (Goldstone et al., 2018), and these can be considered within a pure behavioral perspective or incorporated as part of mentalism and cognition. Indeed, some authors have suggested that this functional-analytic approach could be fruitfully incorporated into a cognitive account, once cognitive phenomena are conceived of as complex environment-behavior relations that are mediated by information processing (Liefooghe and De Houwer, 2016; De Houwer et al., 2018).

This review and conceptual paper sought to outline and explore the various approaches cognitive psychologists have used to study the influence of background knowledge on categorization processes. In doing this it was recognized that although much progress had been made in relation to identifying how background knowledge may affect a task such a when presenting congruent vs. incongruent information, induction, and similarity approaches, much progress was still needed. As such, the current review points to further uses of RFT, to provide some perspective on how to address the knowledge section problem, by expanding on some of the work developed in terms of exemplar, relational induction, and contextual inferences in learning categories.

Naturally, the development of this relationship would be assisted by work such as formalizing a mathematical account (as we have done) of this by incorporating aspects of more traditional cognitive approaches such as connectionist, and induction models. This may be fruitful as both approaches are specialized in different ways, categorization for similarity, rules, and induction, and RFT for modeling functional contextual properties, derived relations, and strength of associations. Future work should now explicitly test implementation of the RFT model specified and within the context of background knowledge. We suggest that attention should be made to some of the novel methodological approaches to test context and situations where RFT properties (e.g., ToF, ME, and CE) arise such as through the clustering approaches suggested (SOM and the deep learning network). This work represents some exciting opportunities for the formal representation of background knowledge, and new insights into how to address the knowledge selection problem, through applying various approaches and levels of heuristics (cognitive and behavioral). It also offers some novel avenues to help facilitate novel approaches in the AGI literature for accounting for crucial background knowledge and broadens existing work in semantic theories of general knowledge.

There are of course some limitations with the mentioned DNN connectionist approach. We explored the use of a biologically plausible network based on Hebbian learning instead of backpropagation (which is not biologically plausible). However, though this is closer to how real neurons learn within the neocortex and other brain areas when compared to standard backpropagation methods, more research should be conducted which ensures that these networks are able to capture the limitations of the human cognitive system (or behavioral learning) when modeling human learning performance.

Finally, the approaches within this modeling, could have some potential clinical applications in the form of process based therapy (PBT) work (Hayes and Hofmann, 2017, 2018; Hofmann and Hayes, 2019). As this models relations of relational networks within RFT and the HDML framework, it could help formulate visual graphs of community networks (as depicted in Figure 10) whereby relational networks could then be integrated and mathematically modeled further within the PBT work. This, therefore, could help researchers and clinicians design complex prediction models which relate RFT with background knowledge in categorization, to the structural equation modeling of PBT (Hofmann et al., 2021), and potentially could have important clinical implications.

## Author Contributions

DE designed and wrote the manuscript. YB-H and CM helped with the RFT articulation and provided some conceptual assistance. The mathematical equations and mathematical interpretation, as well as the illustrative figures, were developed solely by DE. All authors contributed to the article and approved the submitted version.

## Conflict of Interest

The authors declare that the research was conducted in the absence of any commercial or financial relationships that could be construed as a potential conflict of interest.

## Publisher’s Note

All claims expressed in this article are solely those of the authors and do not necessarily represent those of their affiliated organizations, or those of the publisher, the editors and the reviewers. Any product that may be evaluated in this article, or claim that may be made by its manufacturer, is not guaranteed or endorsed by the publisher.

## Footnotes

1 See (Edwards, 2021) for full details of this extended reinforcement framework.

2 See https://neo4j.com/
